# Genome-wide identification of drought-responsive microRNAs in two sets of *Malus* from interspecific hybrid progenies

**DOI:** 10.1038/s41438-019-0157-z

**Published:** 2019-06-08

**Authors:** Chundong Niu, Haiyan Li, Lijuan Jiang, Mingjia Yan, Cuiying Li, Dali Geng, Yinpeng Xie, Yan Yan, Xiaoxia Shen, Pengxiang Chen, Jun Dong, Fengwang Ma, Qingmei Guan

**Affiliations:** State Key Laboratory of Crop Stress Biology for Arid Areas/Shaanxi Key Laboratory of Apple, College of Horticulture, Northwest A&F University, Yangling, Shaanxi 712100 China

**Keywords:** RNA sequencing, Drought

## Abstract

Drought stress can negatively impact apple fruit quality and yield. Apple microRNAs (miRNAs) participate in apple tree and fruit development, as well as in biotic stress tolerance; however, it is largely unknown whether these molecules are involved in the drought response. To identify drought-responsive miRNAs in *Malus*, we first examined the drought stress tolerance of ten F_1_ progenies of R3 (*M*. × *domestica*) × *M. sieversii*. We performed Illumina sequencing on pooled total RNA from both drought-tolerant and drought-sensitive plants. The sequencing results identified a total of 206 known miRNAs and 253 candidate novel miRNAs from drought-tolerant plants and drought-sensitive plants under control or drought conditions. We identified 67 miRNAs that were differentially expressed in drought-tolerant plants compared with drought-sensitive plants under drought conditions. Under drought stress, 61 and 35 miRNAs were differentially expressed in drought-tolerant and drought-sensitive plants, respectively. We determined the expression levels of seven out of eight miRNAs by stem-loop qPCR analysis. We also predicted the target genes of all differentially expressed miRNAs and identified the expression of some genes. Gene Ontology analyses indicated that the target genes were mainly involved in stimulus response and cellular and metabolic processes. Finally, we confirmed roles of two miRNAs in apple response to mannitol. Our results reveal candidate miRNAs and their associated mRNAs that could be targeted for improving drought tolerance in *Malus* species, thus providing a foundation for understanding the molecular networks involved in the response of apple trees to drought stress.

## Introduction

MicroRNAs (miRNAs) are 20–24 nt endogenous small RNAs that silence or downregulate gene expression at the transcriptional and posttranscriptional levels by targeting mRNAs through imperfect sequence complementarity^1–3^. Typically, plant miRNAs are cleaved from hairpin loop precursors by DICER-like1^4^. Increasing evidence suggests that miRNAs are important regulators in various plant processes, such as development^5^ and both biotic and abiotic stress responses^6^.

A variety of approaches, such as northern blotting, cDNA microarrays, and small RNA high-throughput sequencing, have identified miRNA expression patterns in response to drought in *Arabidopsis*^7–9^, rice^10–12^, *Populus trichocarpa*^13,14^, tomato^15^, and cotton^16^. In *Arabidopsis*, northern blotting methods have revealed miRNAs that are responsible for drought-responsiveness. For example, miR169 is downregulated by drought stress^9^. A microarray platform identified 30 miRNAs in rice leaves that are significantly up- or downregulated in response to drought stress^10^, whereas small RNA high-throughput sequencing identified 688 and 155 miRNAs differentially expressed under drought and salt stress in tomatoes^15^ and cotton^16^, respectively. Notably, in different plant species, identical miRNA sequences can have different expression patterns in response to drought. For example, miR408 is downregulated in *Arabidopsis*^17^ but upregulated in *Medicago truncatula*^18^ under drought conditions.

The regulation of miRNAs by drought is multifactorial and manifests across a range of physiological and biochemical mechanisms^19^. Physiological changes, such as early flowering, can improve plant drought resistance^20^. The SQUAMOSA promoter binding protein-like (SPL) transcription factor plays an important role in plant phase transitions and in tissue and architecture development^21^, such as the flowering process. Accordingly, high levels of miR156, which targets the SPL transcription factor, delay flowering time in *Arabidopsis*^22^. Biological processes, such as auxin signaling, ABA-mediated regulation, osmo-protectant biosynthesis, and antioxidant scavenging, also modulate miRNA responses to drought^23^. In *Arabidopsis*, ABA treatment induces the expression of miR393, miR397b, and miR402^7^. Silencing mitogen-activated protein kinase, a target of miR168, impairs ABA and hydrogen peroxide (H_2_O_2_) signaling, resulting in reduced drought tolerance in the tomato plant^24^.

Apple (*Malus* × *domestica* Borkh.) is one of the most commercially important tree fruits worldwide and is mainly propagated through grafting; however, apple trees are often damaged by frequent environmental stressors, such as drought. Drought stress negatively affects fruit quality and yield, and even orchard ecosystem integrity, in some cases^25^. Therefore, it is a high priority to understand the molecular mechanisms orchestrating the response of apple to drought. To date, numerous miRNAs have been identified in apples and associated with either the development of apple trees^26,27^ or fruit^28^, or biotic stress responses^29–31^ but not with drought responses.

*Malus sieversii* is a widely used drought-resistant vigorous rootstock in northwest China^32^, whereas R3 is a promising dwarfing rootstock for apple. However, R3 is drought sensitive. To develop drought-tolerant dwarfing rootstocks for apple, we performed interspecific hybridization between R3 and *M. sieversii*. This approach has been used for breeding disease-resistant apples^33^.

To identify drought-responsive apple miRNAs, we first assessed the drought tolerance of ten F_1_ progenies of R3 (*M*. × *domestica*) × *M. sieversii* and then conducted small RNA sequencing (RNA-seq) on selected drought-tolerant and drought-sensitive plants under control or drought conditions. We provide basic information describing how miRNAs in *Malus* respond to drought stress and this information is urgently needed to facilitate drought tolerance in *Malus* species by genetic engineering methods.

## Materials and methods

### Plant materials and drought stress treatments

All plants were grown at Northwest A&F University, Yangling, China (34°20′N, 108°24′E).

In 2014, 500 interspecific crossed F_1_ progenies of R3 (*M*. × *domestica*, a dwarfing rootstock with a drought-sensitive phenotype) × *M. sieversii* (a vigorous rootstock with a drought-tolerant phenotype) were grown for 4 months in pots containing a local 5:1 (v:v) mixture of loess and sand. We then imposed a drought treatment on the plants for 7 days in mid-July 2014, with mostly sunny weather conditions and a mean high temperature of 34 °C. To avoid the death of F_1_ plants due to drought stress, all F_1_ plants were treated with relatively mild drought conditions. At the end of the drought treatment (where soil moisture content was ~40%), we evaluated the leaf wilting rates (the number of wilting leaves among total leaves in one tree). The wilted leaves were dehydrated and could not be recovered by watering. Plants with wilting rates >50% were classified as drought-sensitive plants.

In 2015, 20 progenies from drought-sensitive and drought-tolerant plants were propagated through tissue culture. Briefly, the buds were disinfected with 75% alcohol, followed by 0.10% mercuric chloride before being placed on Murashige–Skoog (MS) medium supplemented with naphthyl acetic acid, polyvinylpolypyrrolidone, 6-benzylaminopurine (6-BA), indole butyric acid, and sugar. Tissue-cultured F_1_ progenies were propagated and rooted before transplantation to a greenhouse in pots containing a local 5:1 (v:v) mixture of loess and sand. After removing the weak plants, we imposed a drought stress treatment in 2016 on the remaining plants to further confirm phenotypes under drought stress (Table 1). We began treating 10-month-old F_1_ progenies with drought stress on 15 July 2016, with mostly cloudy weather conditions and a mean high temperature of 31 °C. Before drought treatment, these plants were all watered normally (1 liter per pot every 2 days). During drought treatment, the control (each progeny has three trees for control) treatment groups were watered every day to ensure a soil moisture content of 70%. The drought treatment groups were weighed every day and the soil moisture content was calculated. After 12 days of severe drought treatment (where soil moisture content was ~30%), we recorded the wilting rate (trees with wilting rates >50% were defined as wilting trees and the proportion of wilting trees among the total trees in each F_1_ progeny was defined as the wilting rate) of each F_1_ progeny. All plants were subsequently re-irrigated and grown for an additional 30 days and we scored the new growth leaves in each F_1_ progeny as a recovery rate. Plants with recovery rates (in one progeny, the ratio of plants with new growth leaves to total plants) <50% were classified as drought-sensitive plants and those with recovery rates >50% were classified as drought-tolerant plants. Ultimately, we validated four drought-sensitive and six drought-tolerant plants from our drought tolerance assessment in 2014 and our determination of wilting and recovery rates in 2016.

**Table 1:** Wilting rate after drought treatment and recovery rate after rehydration

	Line number	Total number of trees	Drought		Rehydration	
			Number of wilting trees	Wilting rate (%)	Number of recovered trees	Recovery rate (%)
Drought-tolerant plants	0–293	10	7	70	7	70
	0–106	10	6	60	10	100
	0–334	9	5	56	9	100
	7–275	9	4	44	5	56
	8–134	9	7	78	8	89
	0–330	7	1	14	7	100
Drought- sensitive plants	0–297	5	5	100	2	40
	0–131	8	8	100	2	25
	1–338	5	4	80	1	20
	8–11	3	3	100	1	33

Wilting rate means the proportion of wilting trees among the total trees in each F_1_ progeny

### Constructing small RNA libraries for high-throughput sequencing

In 2016, leaf samples were collected from drought-sensitive and drought-tolerant plants after drought treatment for 6 days, when the soil moisture content was between 40% and 50%. We collected three leaves per plant (the 3rd, 4th, and 5th leaves from the top of a branch) from each tree under control (each progeny had three trees) or drought treatment (each progeny had at least six trees), except for F_1_ progenies 0–297, 1–338, and 8–11. F_1_ progenies 0–297, 1–338, and 8–11 had 3–5 plants for drought treatment or control. We divided the leaves from each F_1_ progeny into three biological replicates and each biological replicate contained at least one tree either under drought or control treatment. For each F_1_ progeny biological replicate under control or drought treatment, total RNA was extracted using the miRcute miRNA Isolation Kit (TIANGEN, Beijing, China). Total RNA from drought-tolerant and drought-sensitive plants was pooled separately and used in the small RNA library construction. Specifically, equal amounts of total RNA from each progeny with the same phenotype were pooled as a single biological replicate. Three replicates were included for each control and drought-treated sample. RNA degradation and contamination were monitored on 1% agarose gels and RNA integrity was assayed by the Agilent Bioanalyzer 2100 System (Agilent Technologies, CA, USA).

A total of 3 μg of total RNA per pooled sample was used for the small RNA library construction using the NEBNext^®^ Multiplex Small RNA Library Prep Set for Illumina^®^ (NEB, MA, USA). A total of 12 libraries were constructed, including three drought-sensitive plants under control conditions (S-C), three drought-tolerant plants under control conditions (T-C), three drought-sensitive plants under drought-treated conditions (S-D), and three drought-tolerant plants under drought-treated conditions (T-D). Then, we performed single-end sequencing (50 bp) on an Illumina Hiseq 2500 platform (Illumina, CA, USA) and generated 20 million reads per sample at Novogene Bioinformatics Institute (Novogene, Beijing, China). The small RNA-seq data were deposited to NCBI under accession number SRP110881.

### Identification and analysis of known and novel miRNAs

Raw reads were cleaned by removing the reads containing poly A/T/G/C, reads with 5′ adapter contaminants, reads without the 3′ adapter or the insert tag, and low-quality reads. Q20, Q30, and the GC content of the raw data were calculated to analyze the quality of the small RNA libraries and then a certain length range of clean reads was selected for all downstream analyses. The length distribution from 18 to 30 nt was analyzed for clean reads and the reads ranging from 18 to 30 nt were mapped to the domesticated apple reference genome sequence (https://www.ncbi.nlm.nih.gov/genome/?term = malus) by using Bowtie2^34^, with no mismatches allowed. Unmapped sequences were removed from the data.

The alignment of known miRNAs, prediction of novel miRNAs, miRNA family analysis, miRNA counts and normalization, and differential expression analysis were performed according to Li et al.^35^. To predict novel miRNAs, known miRNA tags obtained by mirdeep2^36^ and srna-tools-cli^37^ were removed. To map every unique small RNA to only one annotation, we filtered out tags of ribosomal RNA (rRNA), transfer RNA (tRNA), small nuclear ribonucleic acid (snRNA), and small nucleolar RNA (snoRNA) with the Rfam database (http://rfam.xfam.org/), repeat sequences with the annotation of *Malus*, natural antisense transcript small interfering RNA (NAT-siRNA) with PlantNATsDB (http://bis.zju.edu.cn/pnatdb/), and genes (exon and intron were included to exclude mRNA degradation fragments) with the gene annotation of *Malus*. Finally, the remaining sRNA tags were used to predict novel miRNAs by miREvo^38^ and mirdeep2^36^, and the read counts were counted as described^35^. For the miRNA family analysis, miFam.dat (http://www.mirbase.org/ftp.shtml) was used for known miRNAs and Rfam (http://rfam.sanger.ac.uk/search/) was used for novel miRNAs. We normalized the abundance of miRNAs in each library using transcripts per million^39^. To obtain drought-responsive miRNAs, the miRNA expression under drought treatment was compared with that under control conditions in drought-tolerant or drought-sensitive plants. To identify drought tolerance-related miRNAs, the miRNA expression in drought-tolerant plants was compared with that in drought-sensitive plants under drought treatment or control conditions. The DESeq R package (1.8.3) was used for the differential expression analysis and the threshold for significant differential expression was *P* < 0.05. To draw heatmaps and Venn diagrams, we used OmicShare tools (http://www.omicshare.com/tools).

### Target prediction and GO enrichment analysis

To target candidate mRNAs for drought improvement in apple rootstocks, we selected differentially expressed miRNAs for further study. The target prediction was made through the psRNATarget server (http://plantgrn.noble.org/psRNATarget/) with default parameters of schema V1 (2011 release)^40^ using the differentially expressed miRNAs and domesticated apple RNA database (https://www.ncbi.nlm.nih.gov/genome/?term = malus). To further evaluate the functional response of miRNA-targeted genes to drought stress in apples, we performed a Gene Ontology (GO) enrichment analysis (agriGO, http://systemsbiology.cau.edu.cn/agriGOv2/) to visualize the enriched biological process, molecular function, and cellular component categories^41^. MiRNA-targeted genes that were differentially expressed between both plant groups under drought were used to draw hierarchical graphs by agriGO.

### qRT-PCR validation of miRNAs and their targets

Stem-loop and regular quantitative reverse-transcriptase PCR (qRT-PCR) methods were used to verify the expression of miRNAs and their targets according to MIQE methods^42^, respectively. Briefly, 4 μg of total RNA was used to synthesize first-strand cDNA with the RevertAid First Strand cDNA Synthesis Kit (Thermo Fisher Scientific, MA, USA). The reverse transcription primers for stem-loop qRT-PCR was a mixture of the *MDH* (malate dehydrogenase) reverse primer and miRNA-specific reverse transcription primer^43^. Oligo-dT primers were used in the regular qRT-PCR. All reactions were performed on the CFX96 Real-Time PCR Detection System (Bio-Rad, CA, USA). Primer efficiency was analyzed by diluting 200 ng cDNA to 40, 8, 1.6, and 0.32 ng. The three biological replicates were the same as those used for the small RNA-seq in each experiment. The 2^−ΔΔCt^ method was used to calculate the relative expression of the genes^44^ with *MDH* as a reference. The data were analyzed using GraphPad Prism 5.0 software (GraphPad Software, CA, USA) and Student’s *t*-test was applied to compare two samples. The primers used in this study are listed in Supplementary Table S9.

### Gene transformation in apple callus and osmotic stress treatment

To overexpress miRNAs in apple calli, the miRNA precursor genes (MIR), including sequences located 100–200 bp upstream and downstream of miRNA stem loops, were cloned by PCR from F_1_ progenies of R3 (*M*. × *domestica*) × *M. sieversii* and then introduced into the pK2GW7 binary vector by Gateway technology (Invitrogen, CA, USA). The primers used for constructing these vectors are shown in Supplementary Table S9. Wild-type (WT) “Orin” apple calli were transformed by *Agrobacterium*-mediated transformation (strain EHA105)^45^. Transgenic calli harboring 35S:MIR156p or 35S:MIRn-249 were screened on MS agar medium (1.5 mg/L 2,4-dichlorophenoxyacetic acid (2,4-D), 0.4 mg/L 6-BA, 30 g/L sucrose, and 8 g/L agar) containing 50 mg/L kanamycin and 250 mg/L cefotaxime. After serial subcultures, the transgenic or WT calli were used to detect the expression of miRNAs by stem-loop qRT-PCR analysis.

For osmotic stress tolerance, calli with similar sizes were selected and cultured on MS agar medium with or without 150 mM mannitol. After 3 weeks, the callus fresh weight was measured. For target expression in the transgenic calli, MS liquid medium (1.5 mg/L 2,4-D, 0.4 mg/L 6-BA, and 30 g/L sucrose) with or without 150 mM mannitol for 6 h at 25 °C in a shaker (120 r.p.m.) was used to extract total RNA.

## Results

### Identification of F_1_ plants with different drought stress tolerances

**Fig. 1:**
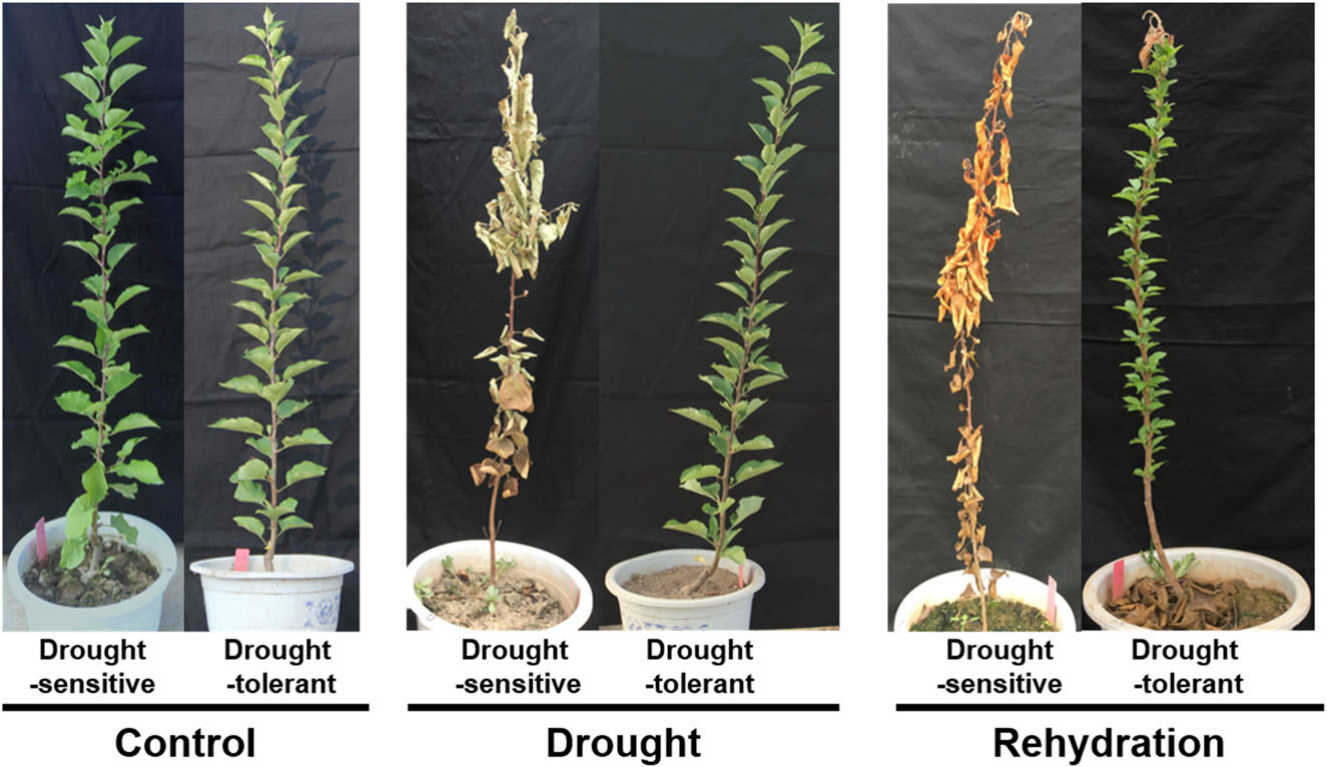
Phenotypes of drought-sensitive and drought-tolerant plants under control (left), drought for 12 days (middle), or rehydration for 30 days (right). The phenotype of F_1_ progenies of R3 (*M.* × *domestica*) × *M. sieversii* is depicted after drought treatment and rehydration. F_1_ progenies with drought-sensitive or drought-tolerant plants were propagated by tissue culture and transplanted to soil for an additional 10 months. The plants were then treated with drought stress for 12 days and rehydrated for 30 days

We identified six F_1_ progenies (0–293, 0–106, 0–334, 7–275, 8–134, and 0–330) as drought-tolerant plants and four (0–297, 0–131, 1–338, and 8–11) as drought-sensitive plants (Fig. 1 and Table 1) based on their recovery rate after rehydration. Plants with recovery rates >50% were defined as drought-tolerant plants and those with recovery rates <50% were defined as drought-sensitive plants.

### Overview of sRNA sequencing

**Fig. 2:**
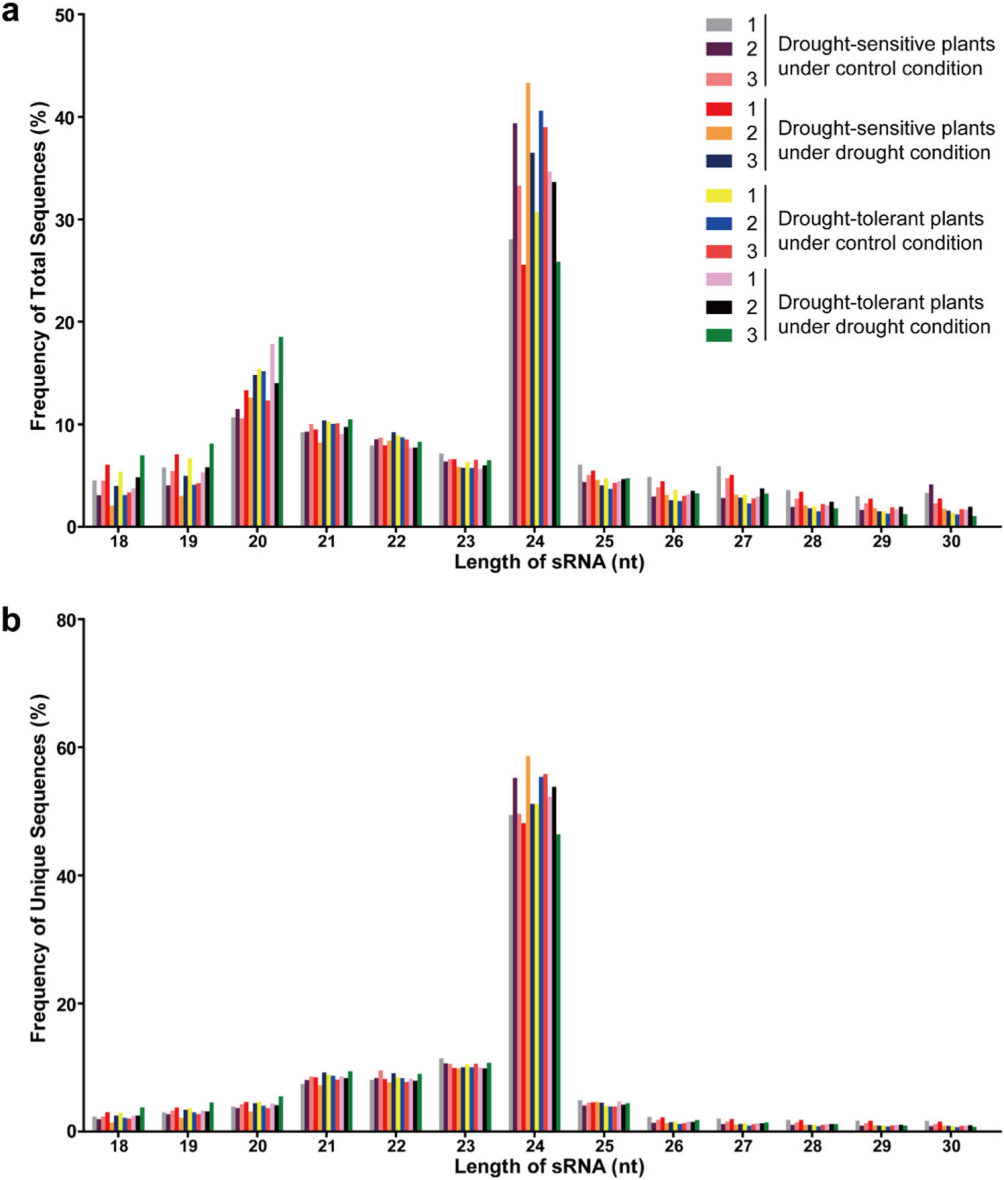
Mapping status of sRNAs in each library. **a** redundant sRNAs. **b** unique sRNAs. The numbers 1, 2, and 3 indicate three biological replicates

Agarose electrophoresis analysis suggested a decent quality of the extracted RNA for each biological replicate of F_1_ progeny under control or drought treatment (Supplementary Fig. S1a). Analysis by agarose electrophoresis and Agilent 2100 also showed a decent quality of the pooled RNA (Supplementary Fig. S1b and c). In addition, the quality of all libraries was good enough for downstream analysis (Supplementary Table S1). We sequenced a total of 368.88 million (M) raw and 356.58 M clean reads, with a mean of 29.71 M (97%) clean reads per library (Table 2). For further analysis, we isolated 264.21 M clean reads with lengths ranging from 18 to 30 nt (74%) and 67.21 M unique reads (25%) (Supplementary Table S1). All libraries had a similar distribution of lengths (~72% for redundant reads and 84% for unique reads, Fig. 2). The 24 nt reads were the most abundant in redundant reads (34%) and unique reads (52%), followed by the 20 nt redundant reads (14%) and 23 nt unique reads (10%) (Fig. 2). Among the 18–30 nt sRNA, 83% redundant reads and 71% unique reads were successfully mapped to the apple reference genome (Supplementary Table S1).

**Table 2:** Overview of miRNA sequencing reads

Sample		Total reads	*N*% > 10%	Low quality	5′-Adapter contamine	3′-Adapter null or insert null	With poly A/T/G/C	Clean reads
Drought-sensitive plants	Control 1	33,928,632 (100%)	371 (0%)	81,721 (0.24%)	33,287 (0.10%)	1,377,648 (4.1%)	53,145 (0.16%)	32,382,460 (95%)
	Control 2	31,905,071 (100%)	360 (0 %)	84,190 (0.26%)	32,884 (0.10%)	555,157 (1.7%)	70,210 (0.22%)	31,162,270 (98%)
	Control 3	33,330,848 (100%)	349 (0%)	77,489 (0.23%)	32,009 (0.10%)	653,356 (2.0%)	58,660 (0.18%)	32,508,985 (98%)
	Drought 1	32,537,917 (100%)	347 (0%)	82,237 (0.25%)	27,717 (0.09%)	875,074 (2.7%)	37,609 (0.12%)	31,514,933 (97%)
	Drought 2	24,779,273 (100%)	252 (0%)	66,295 (0.27%)	25,252 (0.10%)	709,788 (2.9%)	64,004 (0.26%)	23,913,682 (97%)
	Drought 3	30,900,174 (100%)	328 (0%)	75,907 (0.25%)	34,188 (0.11%)	775,123 (2.5%)	67,303 (0.22%)	29,947,325 (97%)
Drought-tolerant plants	Control 1	31,489,922 (100%)	443 (0%)	86,581 (0.27%)	28,714 (0.090%)	1,352,490 (4.3%)	39,785 (0.13%)	29,981,909 (95%)
	Control 2	29,888,571 (100%)	430 (0%)	78,512 (0.26%)	32,389 (0.11%)	855,209 (2.9%)	58,814 (0.20%)	28,863,217 (97%)
	Control 3	26,866,676 (100%)	435 (0%)	73,980 (0.28%)	24,177 (0.090%)	448,026 (1.7%)	62,656 (0.23%)	26,257,402 (98%)
	Drought 1	29,371,046 (100%)	444 (0%)	73,889 (0.25%)	31,098 (0.11%)	504,062 (1.7%)	51,860 (0.18%)	28,709,693 (98%)
	Drought 2	30,422,600 (100%)	445 (0%)	85,569 (0.28%)	26,499 (0.090%)	978,522 (3.2%)	45,285 (0.15%)	29,286,280 (96%)
	Drought 3	33,456,352 (100%)	479 (0%)	82,842 (0.25%)	28,253 (0.080%)	1,266,189 (3.8%)	29,138 (0.090%)	32,049,451 (96%)

### Identification of known and novel miRNAs in apple

A total of 4.4 M redundant reads, including 205 precursors and 206 mature miRNAs, were mapped to the miRbase without mismatch (Supplementary Tables S2 and S3).

To identify novel miRNAs in apple plants, a total of 1.3 M redundant reads (0.57%) were mapped onto 274 hairpin structures, identifying 253 candidates, among which 123 had miRNA* sequences (Supplementary Tables S2 and S4). As miRNA* is a common tool to verify new miRNAs^46^, we designated the 123 candidates with miRNA* sequences as novel miRNAs and the remaining 130 without miRNA* sequences as novel miRNA candidates. The lengths of the 253 novel apple miRNAs ranged from 18 to 24 nt, with a dominant distribution of 24 nt (211 miRNAs), followed by 21 nt (18 miRNAs), 22 nt (12 miRNAs), 23 nt (4 miRNAs), 18 nt (3 miRNAs), 19 nt (3 miRNAs), and 20 nt (2 miRNAs) (Supplementary Table S4).

**Fig. 3:**
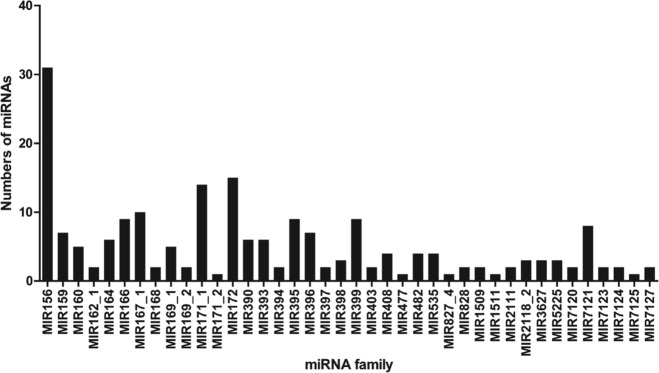
Number of identified miRNAs in known miRNA families

The identified known and novel miRNAs were classified into 40 families. Among these families, the largest was miR156, with 31 members, followed by miR172 (15 members), miR171_1 (14 members), and miR167_1 (10 members). Only two novel miRNAs were classified into miRNA families: miRn234 into the miR159 family and miRn85 into the miR169_2 family (Fig. 3 and Supplementary Table S5).

In addition to the known and novel miRNA tags, small RNA reads were also mapped to rRNA, tRNA, snRNA, snoRNA, repeat sequences, NAT-siRNA, and genes. The most abundant redundant reads were mapped onto genes (17%), followed by repeat sequences (16%), rRNAs (8.1%), and NAT-siRNAs (7.2%). Unique reads were also most abundant in repeat sequences (26%), followed by genes (7.8%), rRNAs (1.3%), and NAT-siRNAs (1.2%). Among unique and redundant reads, the minimum RNA classes were snoRNA (0.11% and 0.22%, respectively), snRNA (0.080% and 0.10%, respectively), and tRNA (nearly 0% for both) (Supplementary Table S2).

### Expression analyses of the miRNAs in apple

**Fig. 4:**
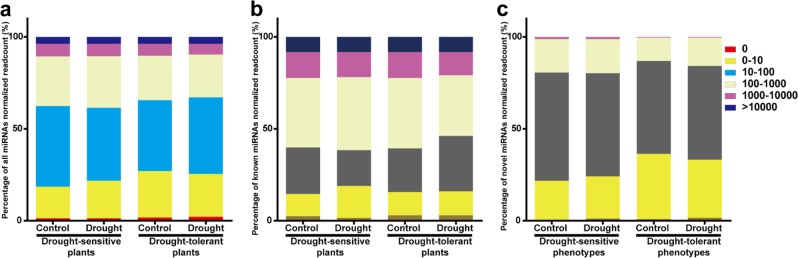
Expression levels of miRNAs with normalized read count frequencies in each treatment. **a** all identified (including known and novel) miRNAs. **b** known miRNAs. **c** novel miRNAs. Expression level category: 0, no expression; 0–10, extra-low expression; 10–100, low expression; 100–1000, moderate expression; 1000–10,000, high expression; >10,000, extra-high expression

The miRNAs identified in drought-sensitive or drought-tolerant plants in each treatment were classified into six categories of expression based on normalized read counts: no (0 reads), extra-low (0–10 reads), low (10–100 reads), moderate (100–1000 reads), high (1000–10,000 reads), and extra-high (>10,000 reads) expression (Fig. 4). Highly similar percentages of identified miRNAs fell into the same category in each treatment (Fig. 4a). The largest percentage of known miRNAs fell into the low category, followed by the moderate and extra-low categories, whereas the lowest percentages fell into the no expression category (Fig. 4b and Supplementary Table S6). MiR1511 was the most abundant miRNA, with over 60,000 normalized reads in each treatment. The majority of novel miRNAs had low abundances in each treatment (Fig. 4c and Supplementary Table S6). For example, miRn-54 had 0.75 normalized reads in a library of untreated control drought-tolerant plants. Moreover, different members of the same miRNA families displayed different expression levels. For example, miR156h had more than 300 normalized reads, but miR156t had <10 (Supplementary Table S6). Notably, 20 miRNAs were absent in at least one treatment, including 12 known and 8 novel miRNAs (Supplementary Table S6). In drought-tolerant plants, miR3627 a/b/c, miRn-50, miR-61, and miRn-158 were not present under drought treatment, whereas miR399 e/f/g/h, miRn-67, and miRn-203 were not detected under control conditions. In drought-sensitive plants, miR828a and miR828b were absent under both conditions, whereas miRn-11 and miRn-78 were not present under drought. In addition, miR169c and miR169d were only detected in drought-sensitive plants under drought, whereas miR7128 and miRn-54 were only detected in drought-tolerant plants under control conditions (Supplementary Table S6).

**Fig. 5:**
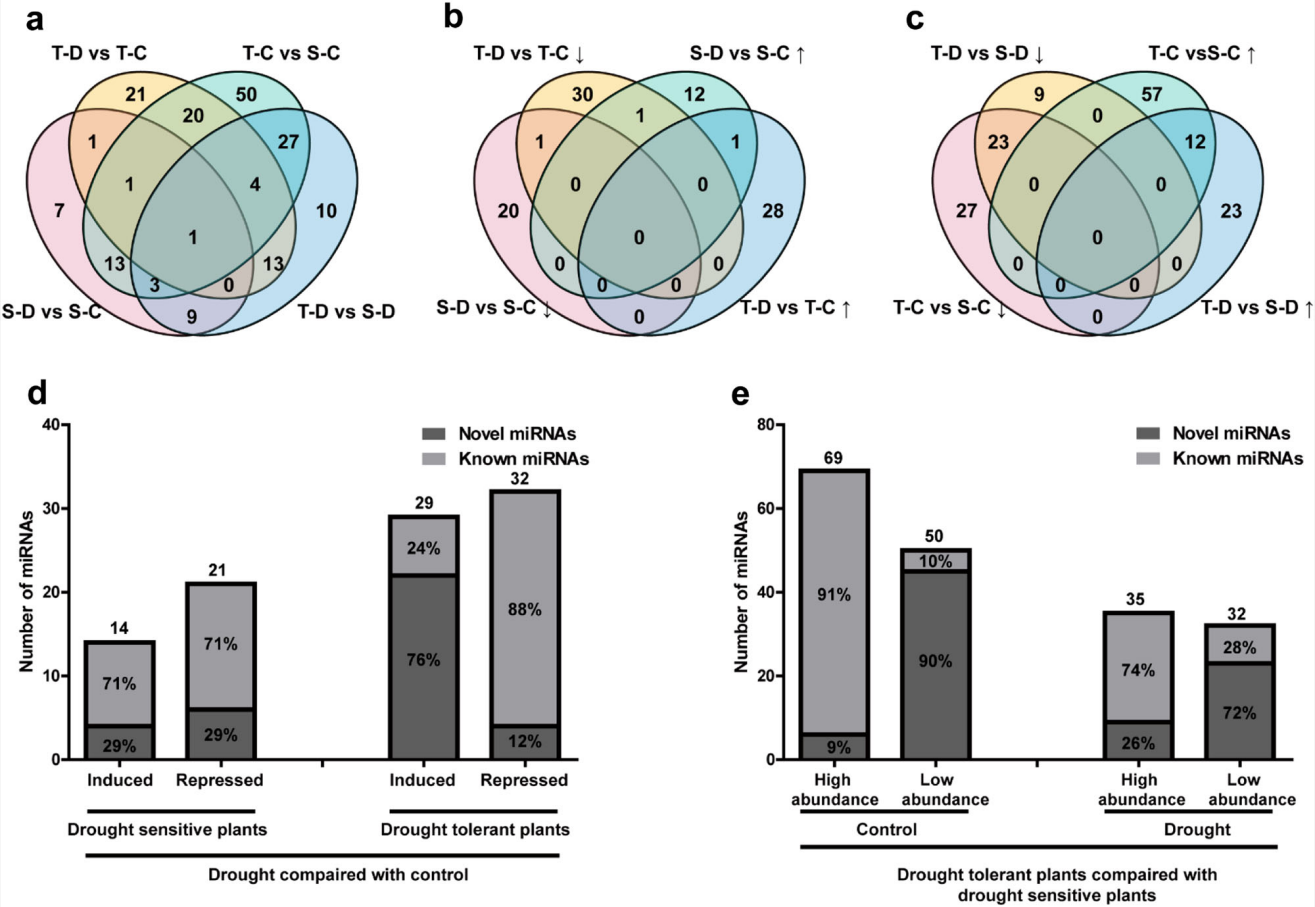
Diagrams of differentially expressed miRNAs. **a** Distribution of miRNA differences among the four comparison groups. **b** Differentially expressed miRNAs induced or repressed by drought. **c** Differentially expressed miRNAs in drought-tolerant vs. drought-sensitive plants. **d** Drought-induced or -repressed novel and known miRNAs in both phenotypes. **e** High abundant or low abundant miRNAs in drought-tolerant plants under drought treatment or control conditions compared with drought-sensitive plants. S-C: drought-sensitive plants under control conditions; S-D: drought-sensitive plants under drought treatment; T-C: drought-tolerant plants under control; T-D: drought-tolerant plants under drought treatment. Upward arrows indicate upregulation or higher abundance and downward arrows indicate downregulation or lower abundance

**Fig. 6:**
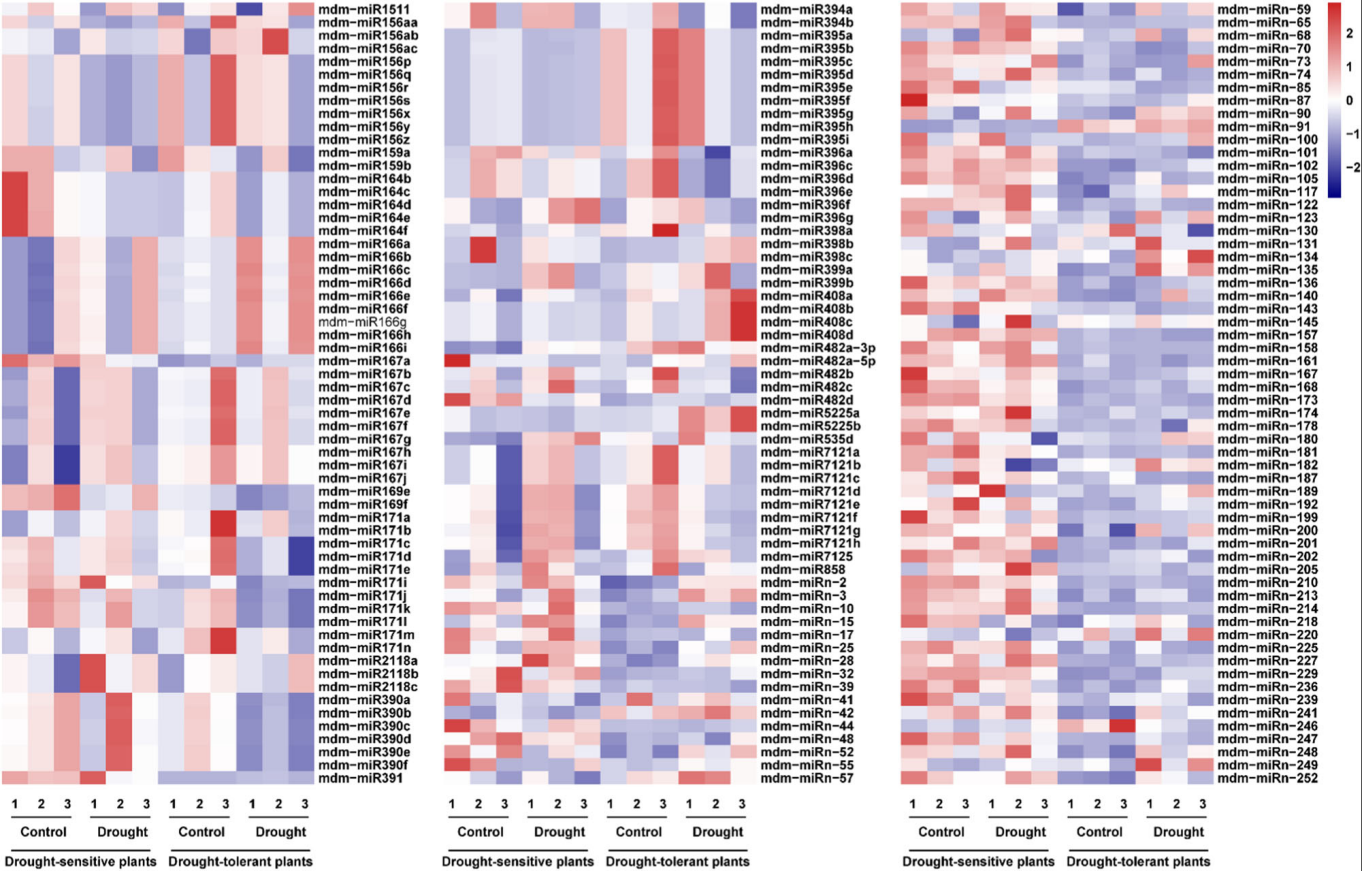
Heat map analysis of differentially expressed miRNAs in four groups. Up- and downregulated genes are indicated in red and blue, respectively. Color brightness reflects the magnitude of difference. The numbers 1, 2, and 3 indicate three biological replicates

The expression levels of miRNAs in drought-tolerant or drought-sensitive plants under control or drought treatment are provided in Figs. 5 and 6, and Supplementary Table S7. Thirty-nine percent of miRNAs displayed significant expression levels (*p*-value < 0.05) and 93 differentially expressed miRNAs were identified in the drought response. After drought treatment, 21 miRNAs were downregulated and 14 miRNAs were upregulated in drought-sensitive plants, whereas 32 miRNAs were downregulated and 29 miRNAs were upregulated in drought-tolerant plants (Fig. 5a, b). Among these 93 differentially expressed miRNAs, only 3 novel miRNAs (miRn15, miRn85, and miRn246) were responsive to drought in both plant groups (Fig. 6 and Supplementary Table S7). Specifically, miRn15 was upregulated and miRn85 was downregulated in both plants in response to drought, whereas miRn246 was downregulated in drought-tolerant plants but upregulated in drought-sensitive plants in response to drought. Moreover, drought treatment induced most novel miRNAs (76%) and repressed most known miRNAs (88%) in drought-tolerant plants, but upregulated (71%) and downregulated (71%) most known miRNAs in drought-sensitive plants (Fig. 5d and Supplementary Table S7).

When comparing drought-sensitive and drought-tolerant plants, 151 miRNAs were differentially expressed under treatment, including 119 miRNAs under control and 67 miRNAs under drought (Fig. 5a, c). These 67 differentially expressed miRNAs represent a unique response of miRNAs in drought-tolerant plants and may be important miRNAs for improving the drought tolerance of apple rootstocks. Among these 67 miRNAs, 35 were differentially expressed under both normal and drought conditions, including 23 lower abundance and 12 higher abundance miRNAs in drought-tolerant plants (Fig. 5c). Among these miRNAs, miRn-157, miRn-158, and miRn-101 were classified as lower abundance in drought-sensitive plants, whereas miR395 a/b/c/d/e/f/g/h/i were classified as higher abundance (Supplementary Table S7).

In addition, the expression levels of 22 miRNAs that were similar in both plant groups under normal conditions were either reduced or increased after drought treatment, including miR156 aa/p/q/r/s/x/y/z, miR390 a/b/c/d/e/f, miRn-134, miRn-249, and miR5225 a/b (Supplementary Table S7). Of these 22 miRNAs, nine were responsive to drought in drought-sensitive plants and 13 were responsive to drought in drought-tolerant plants (Figs. 5a and 6).

Under control conditions, 69 miRNAs displayed higher abundance in drought-tolerant plants, whereas 50 miRNAs displayed lower abundance in drought-tolerant plants (Fig. 5c). In drought-tolerant plants relative to drought-sensitive plants, 35 miRNAs were higher in abundance, whereas 32 miRNAs were lower in abundance after drought treatment (Fig. 5c). Under both normal and drought conditions, most known miRNAs were more abundant (91% in control and 74% in drought treatment), whereas most novel miRNAs were less abundant (90% in control and 72% in drought treatment) in drought-tolerant plants than in drought-sensitive plants (Fig. 5e and Supplementary Table S7).

### Target prediction and GO analysis

We predicted 2754 miRNA target pairs, including 1090 unique protein-coding mRNAs. Among the 1090 protein-coding mRNAs, the majority of genes encoded transcription factors (SPL transcription factors, C3HC4-type RING finger), resistance-associated proteins (TIR-NBS-LRR class disease resistance protein), transport proteins (sulfate transporter protein), and enzymes, such as kinases (receptor kinase), transferases (*S*-adenosyl-l-methionine-dependent methyltransferases), and phosphatases (RNA polymerase II C-terminal domain phosphatase) (Supplementary Table S8).

**Fig. 7:**
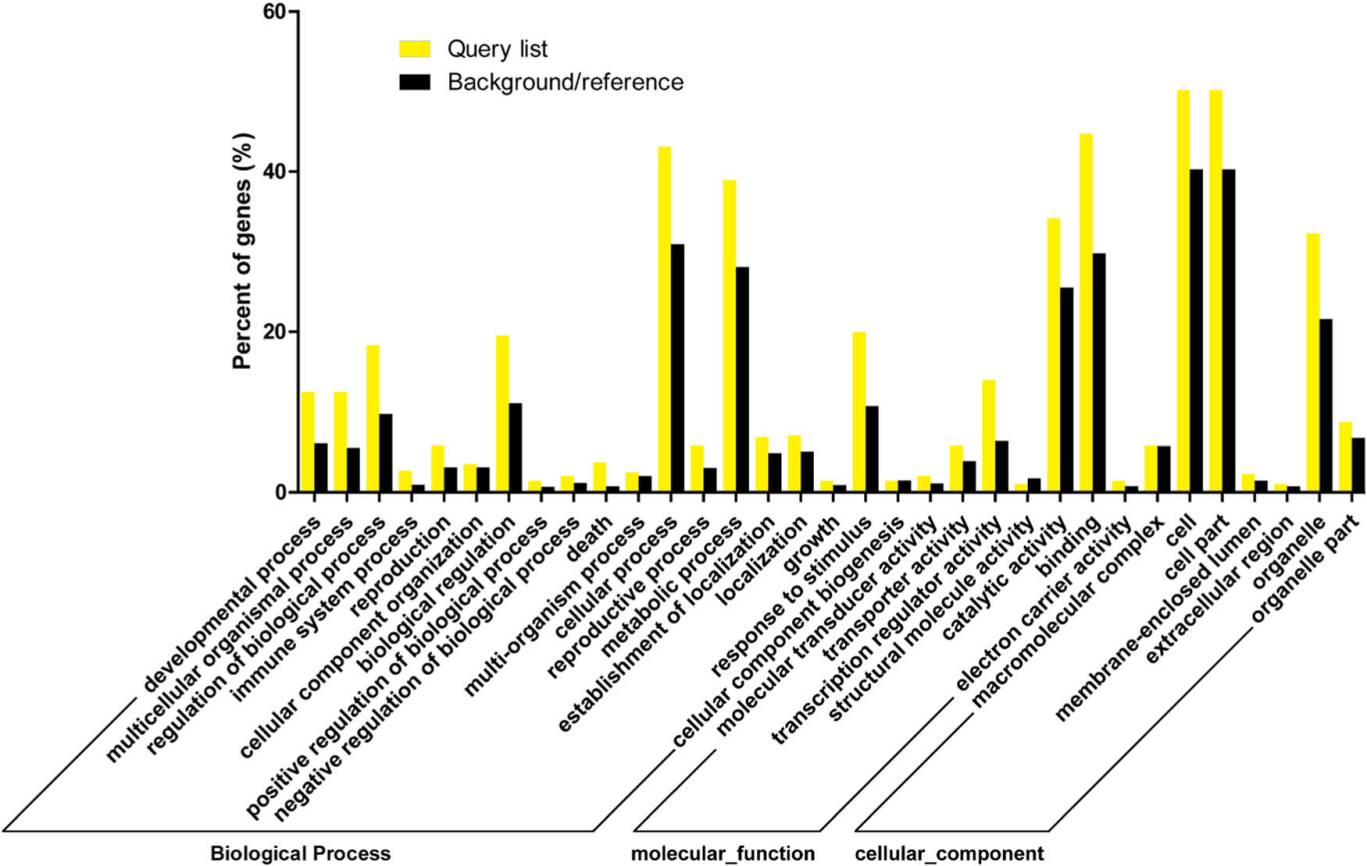
GO enrichment analysis of the target gene candidates of differentially expressed miRNAs. The *x*-axis is the GO term targeting gene enrichment, whereas the *y*-axis represents the percentage of target genes mapped by the term. The query list (yellow bar) represents the percentage of target genes mapped to the GO term in all genes of the input list. The background/reference list (dark bar) represents the percentage of all GO term genes from all reference database genes

GO analysis of 1090 protein-coding mRNAs suggested that enriched biological processes included cellular process (GO:0009987), metabolic process (GO:0008152), and response to stimulus (GO:0050896), whereas binding (GO:0005488) and catalytic activity (GO:0003824) were the most abundant classes in the molecular function category (Fig. 7). The most significant cellular components were cell (GO:0005623), cell part (GO:0044464), and organelle (GO:0043226).

**Fig. 8:**
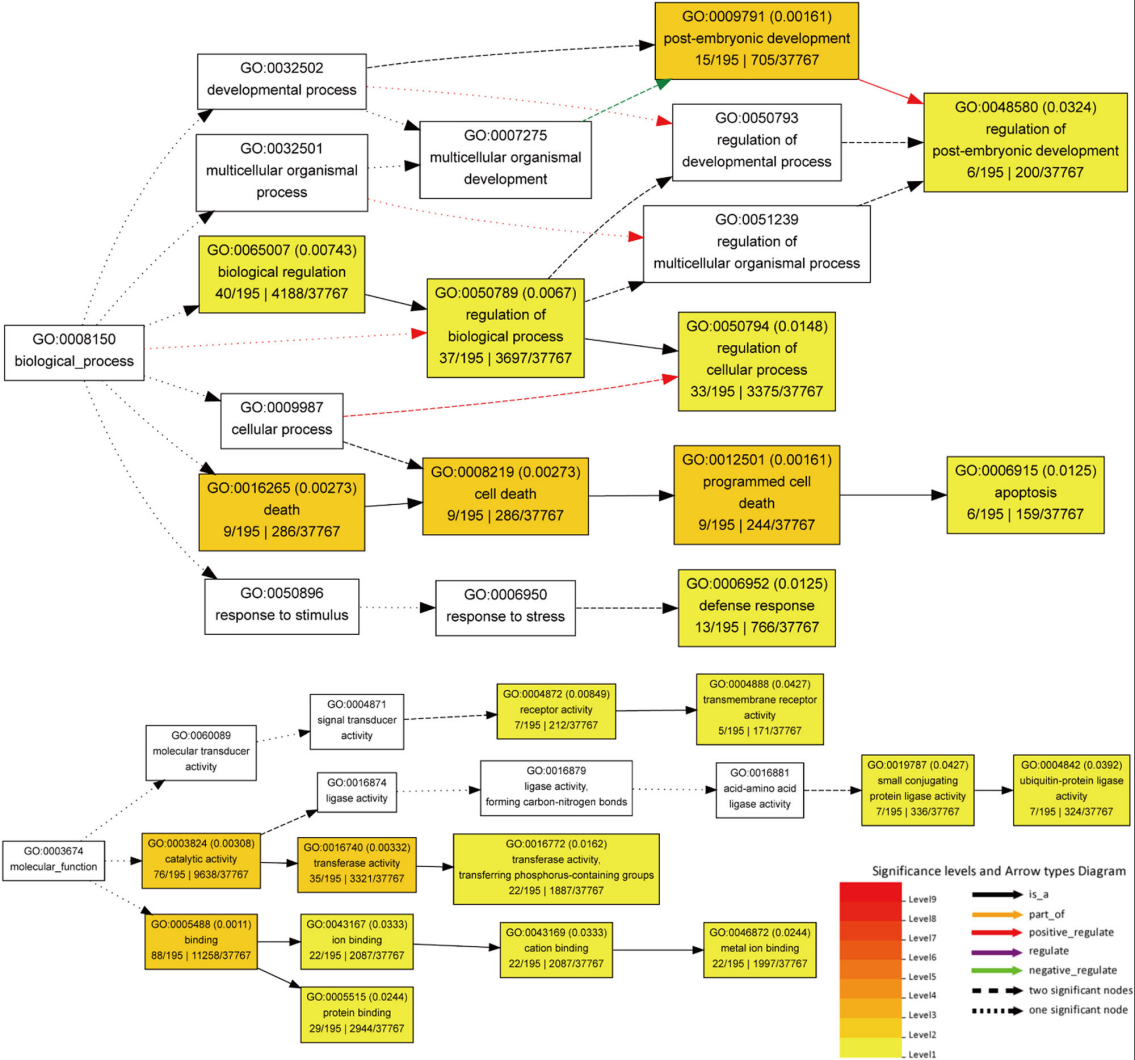
Diagrams of enriched GO terms of target genes constructed by agriGO. Target genes of 67 differentially expressed miRNAs in the drought-tolerant plants compared with the drought-sensitive plants under drought were used in agriGO analysis. Significant terms are indicated in red, with darker shades reflecting greater significance

We also evaluated the potential functions of 67 miRNA-targeted genes that were differentially expressed between both plant groups under drought. We selected a total of 1213 miRNA target pairs for GO enrichment analysis, including 390 protein-coding mRNAs. Hierarchical graphs displayed significantly enriched GO terms in biological process and molecular function categories, rather than the cellular component. Within the biological process category, programmed cell death (GO:0012501), defense response (GO:0006952), postembryonic development (GO:0009791), regulation of cellular process (GO:0050794), and biological regulation (GO:0065007) were significantly enriched (Fig. 8). Within the molecular function terms, receptor activity (GO: 0004872), ubiquitin-protein ligase activity (GO: 0004842), transferase activity (GO:0016740), ion binding (GO:0043167), and protein binding (GO: 0005515) were significantly enriched (Fig. 8).

### Expression verification of miRNAs and their targets by qRT-PCR

**Fig. 9:**
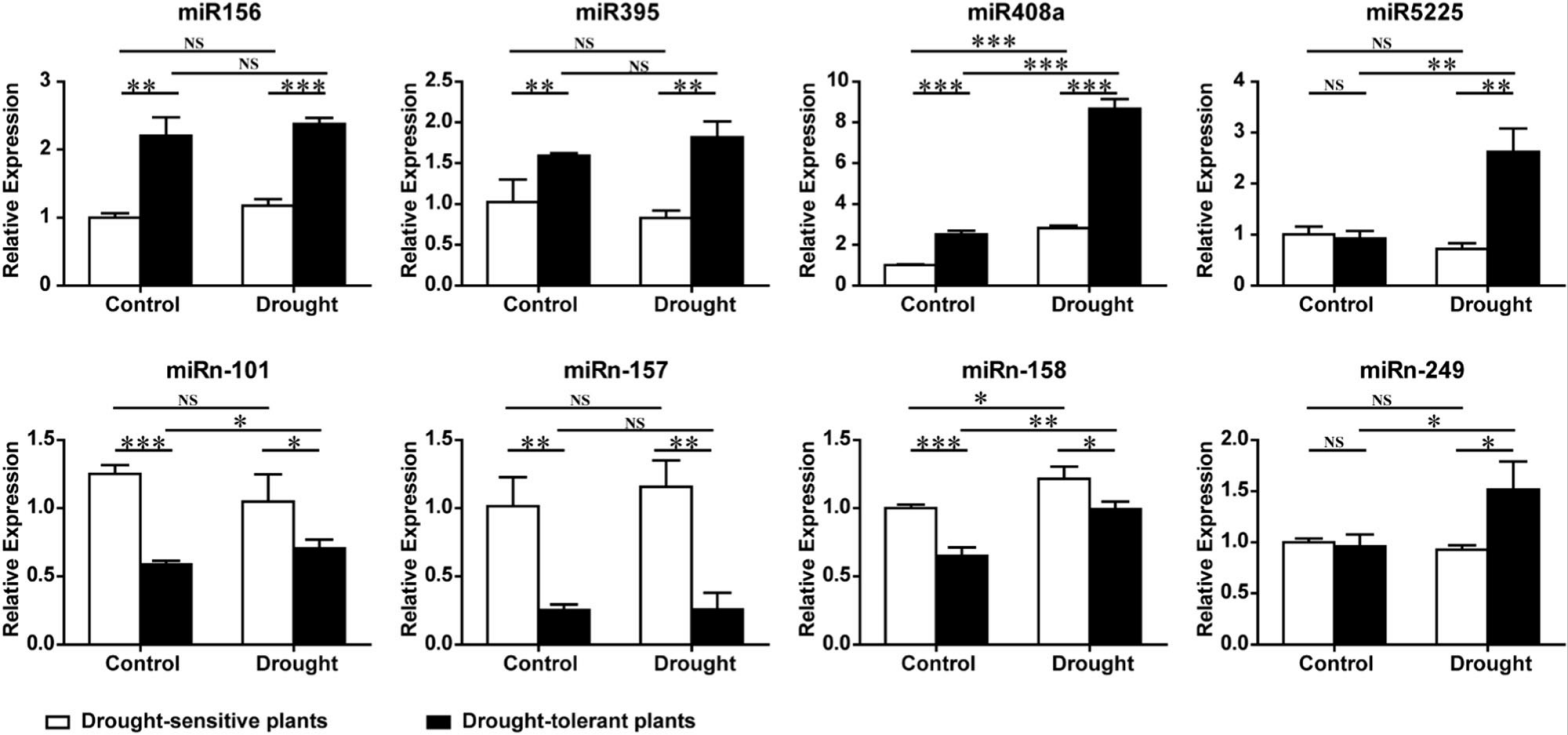
Verification of differentially expressed known and novel miRNAs by stem-loop qRT-PCR analysis. The expression of four known miRNAs (miR156, miR395, miR408a, and miR5225) and four novel miRNAs (miRn-101, miRn-157, miRn-158, and miRn-249) was verified by stem-loop qRT-PCR, with *MDH* (malate dehydrogenase) as an internal reference gene. *MDH* reverse primer and miRNA-specific reverse transcription primer were mixed, and the mixed primer and total RNA (same as sRNA-seq) were used for cDNA synthesis. Error bars indicate ±SD (*n* = 3, from three technical replicates). Three biological replicates were performed. Student’s *t*-test was performed for data analysis and statistically significant differences were indicated by *p-value < 0.05, **p-value < 0.01, and ***p-value < 0.001

We used stem-loop qRT-PCR to validate sequencing results by determining miRNA expression levels in both plant groups under control and drought treatments. From the high-throughput sequencing results, we selected eight miRNAs (four conserved and four novel) for stem-loop qRT-PCR, including miR156, miR395, miR408a, miR5225, miRn-101, miRn-157, miRn158, and miRn-249 (Fig. 9). Primer analysis suggested that all stem-loop qRT-PCR primers had high efficacy and were thus suitable for miRNA expression detection (Supplementary Fig. S2a and b). The stem-loop qRT-PCR results confirmed the expression of seven of the eight miRNAs (with the exception of miR156), indicating the reliability of our small RNA-seq data. The expression of miR156, miR395, and miR408a was much higher in drought-tolerant plants than in drought-sensitive plants under control or drought conditions. MiR408a expression was induced by drought in both plant groups, whereas miR156 and miR395 were not. Under control conditions, the expression of miR5225 and miRn-249 was similar between drought-tolerant and drought-sensitive plants; however, under drought conditions, the increased expression of both miRNAs in drought-tolerant plants was observed. In addition, the expression of both miR5225 and miRn-249 was only induced by drought in drought-tolerant plants. MiRn-101, miRn-157, and miRn-158 had similar expression patterns with lower expression in drought-tolerant plants under both control and drought conditions (Fig. 9). The expression pattern of miR156 by stem-loop qRT-PCR was not consistent with the small RNA-seq results, possibly because stem-loop qRT-PCR detected all miR156 family members (Figs 6 and 9).

**Fig. 10:**
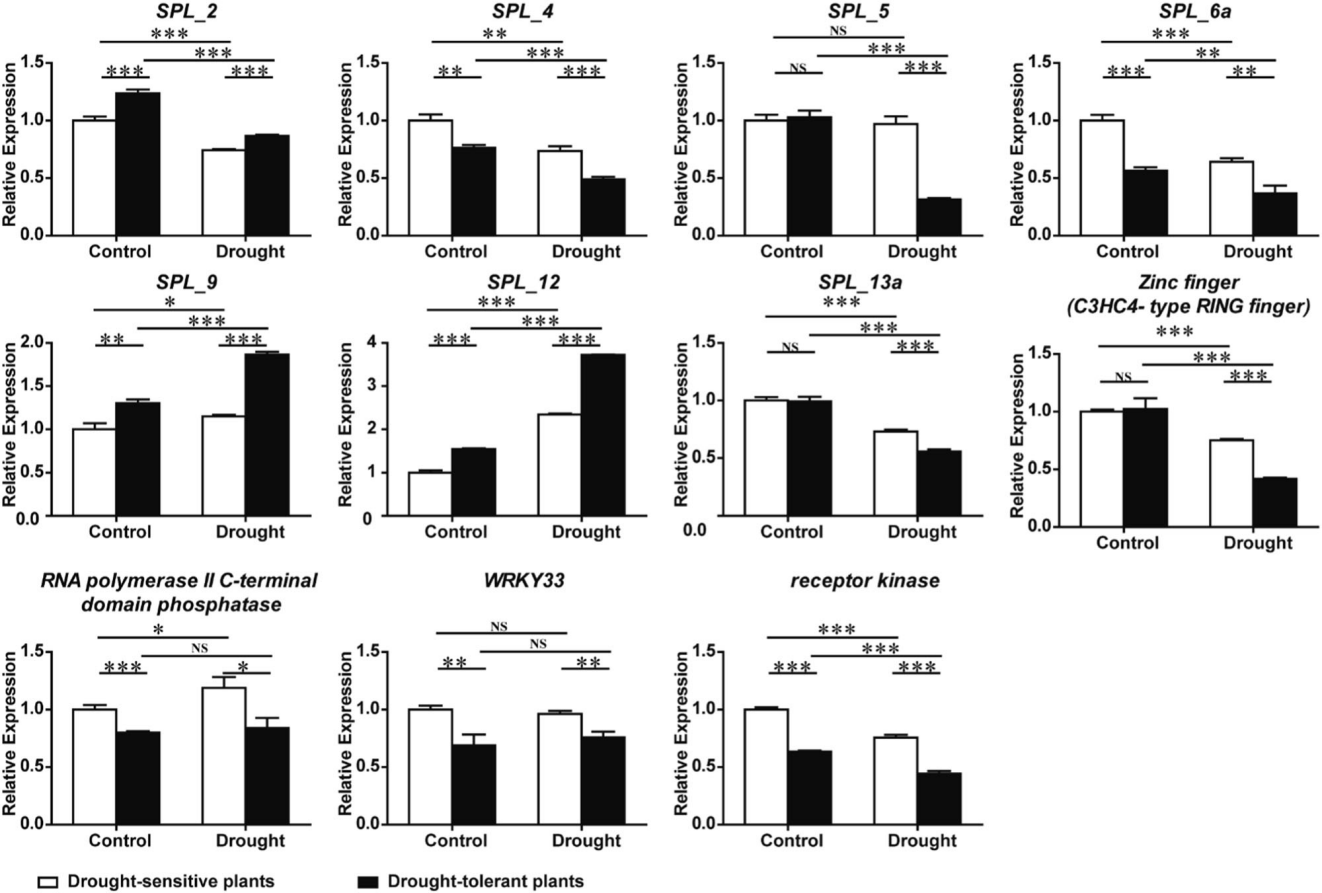
Expression of the targets of differentially expressed miRNAs by qRT-PCR. Eight miR156 target genes (*SPL_2*, *SPL_4*, *SPL_5*, *SPL_6a*, *SPL_9*, *SPL_12*, and *SPL_13a*), three miR395 target genes (*RNA polymerase II C-terminal domain phosphatase*, *WRKY33*, and receptor kinase), and one novel miRNA (miRn-249) target gene (*Zinc finger* (*C3HC4-type RING finger*)) were analyzed by qRT-PCR. Oligo-dT primers and total RNA (same as sRNA-seq) were used for cDNA synthesis, with *MDH* (malate dehydrogenase) as an internal reference gene. Error bars indicate ±SD (*n* = 3, from three technical replicates). Three biological replicates were performed. Student’s *t*-test was performed for data analysis and statistically significant differences were indicated by *p-value < 0.05, **p-value < 0.01, and ***p-value < 0.001

We next analyzed the expression patterns of miRNA-targeted genes by using high-efficiency primers (Supplementary Fig. S3a and b), including seven miR156 targets, three miR395 targets, and one novel miRNA (miRn-249) targeted gene. The qRT-PCR results demonstrated that three predicted target genes of miR395 (*RNA polymerase II C-terminal domain phosphatase*, *WRKY33*, and *receptor kinase*) had expression patterns opposite that of miR395 in both plant groups under control and drought conditions. This finding indicated a negative correlation with miR395, supporting the potential of these genes as miR395 targets (Fig. 10). *Zinc finger* (*C3HC4-type RING finger*) was predicted as the target of miRn-249 under drought, with an expression pattern that negatively correlated with that of miRn-249 in both plant groups under drought. The expression patterns of *SPL* genes were more complicated. *SPL_4* and *SPL_6a* displayed expression patterns opposite that of miR156 under control or drought, whereas the expression of *SPL_5* and *SPL_13a* was only negatively correlated with that of miR156 transcripts under drought. Thus, both *SPL* genes may only be targeted by miR156 under drought conditions. *SPL_2*, *SPL_9*, and *SPL_12* had the same expression pattern as miR156 under control and drought, indicating that these three genes may not be the targets of miR156 in apple rootstocks (Fig. 10).

### Function verification of miRNAs in response to osmotic stress

**Fig. 11:**
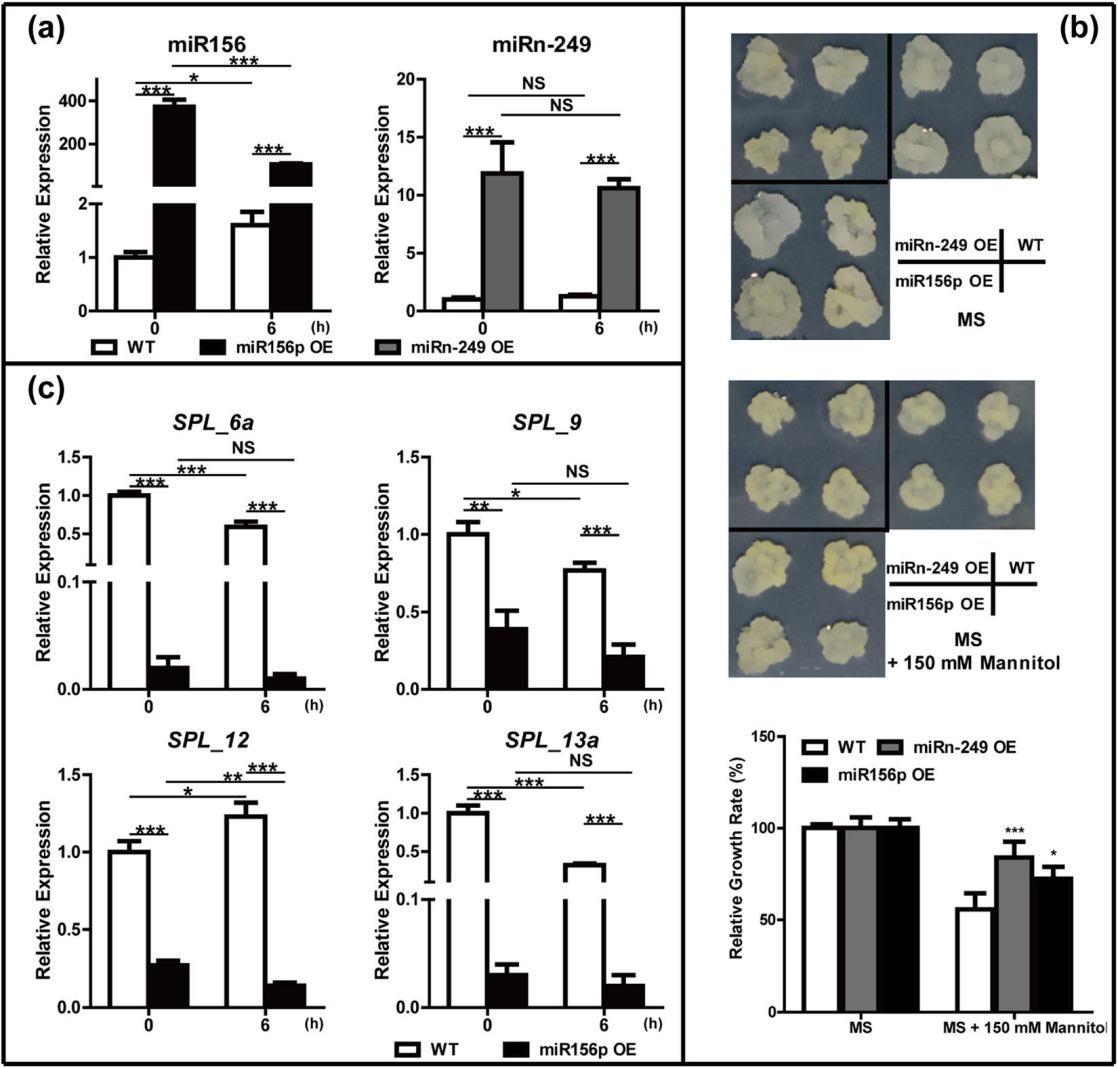
Effect of osmotic stress in callus derived from WT, miR156p-, and miRn-249-overexpressing callus. **a** Expression levels of miR156p or miRn-249 in overexpressing calli under control and 150 mM mannitol treatment for 6 h. **b** Morphology of the calli after culture for 3 weeks on MS medium (upper) or MS medium supplemented with 150 mM mannitol (middle), and relative growth rate between them (under, error bars indicate ±SD, *n* ≥ 10). **c** Expression levels of the miR156p target genes under control and 150 mM mannitol treatment for 6 h. Stem-loop qRT-PCR and qRT-PCR used *MDH* (malate dehydrogenase) as an internal reference gene, error bars indicate ±SD (*n* = 3, from three technical replicates) and three biological replicates were performed. Student’s *t*-test was performed for data, and statistically significant differences were indicated by *p-value < 0.05, **p-value < 0.01, and ***p-value < 0.001

To verify the function of these miRNAs, we selected two miRNAs, miR156p and miRn-249, and generated transgenic calli carrying 35S:MIR156p or 35S:MIRn-249. As drought stress could cause osmotic stress in plants, we treated the WT and transgenic calli with 150 mM mannitol. Stem-loop qRT-PCR analysis demonstrated that miR156p was upregulated ~400-fold in the transgenic calli compared with that in WT calli and 10-fold for miRn-249 in miRn-249 transgenic calli (Fig. 11a). When treated with 150 mM mannitol for 3 weeks, the relative growth rate of miRn-249 and miR156p transgenic calli was higher than that of WT (Fig. 11b), suggesting positive roles for these two miRNAs in osmotic stress tolerance. We also detected the expression levels of the target genes of these two miRNAs. Our results showed that four target genes of miR156 (*SPL_6a*, *SPL_9, SPL_12*, and *SPL_13a*) were repressed by elevated miR156p expression (Fig. 11c). However, the expression of *SPL_2* and *Zinc finger* (*C3HC4-type RING finger*), which were predicted as the targets of miR156 and miRn-249, respectively, was not reduced in miR156p or miRn-249 transgenic calli (Supplementary Fig. S4).

## Discussion

Drought is a significant and common environmental stressor, restricting apple yield and quality worldwide. MiRNAs, the negative regulators of gene expression, are associated with abiotic stress^47,48^, and some drought-responsive miRNAs have been identified in rice^10,12^, *Populus*^13,49^, *Arabidopsis*^7,8^, wheat^50^, maize^51^, soybean^52^, peach^53^, and barley^54^. However, there is no study that systematically identifies and describes the expression dynamics of miRNAs in apple trees in response to drought stress using a deep-sequencing approach. In this study, we identified miRNAs important for the drought tolerance of apple rootstocks by evaluating two pools of F_1_ hybrids with contrary drought phenotypes.

During an interspecific cross, gene recombination occurs; thus, F_1_ progenies from a cross of drought-tolerant rootstock with drought-sensitive rootstock can exhibit differences in drought resistance. Therefore, some drought-resistant genes can be transferred to certain progeny with other desired phenotypes. These genes may play an important role in improving the drought resistance of apple rootstocks. Due to heterozygosity within the apple genome, the genetic background of F_1_ progenies varies from one to another, even though the F_1_ progenies have the same parents. To avoid any discrepancy in the genetic background between drought-tolerant and drought-sensitive plants, we used pooled RNA from two groups of plants: drought-tolerant plants and drought-sensitive plants. This strategy is commonly applied to normalize the genetic background of heterozygous plants^55–57^. We generated 12 small RNA libraries with three biological replicates to strengthen the power of our small RNA-seq results. Indeed, sRNAs from each library shared a similar distribution pattern (Fig. 2), consistent with earlier findings in apple trees^58^. Almost all identified known miRNAs, except miR399c, were matched to the miRbase, suggesting that F_1_ progenies had the same known miRNAs as Golden Delicious, as previously reported^58^. Among these miRNAs, the largest families were consistent with previous research^26^ and the majority of the most abundant miRNAs (such as miR1511, miR166, miR167, and miR396) were conserved across plant species^12^ (Fig. 3).

Many miRNAs are differentially expressed under drought conditions^6^. In our study, 93 miRNAs were determined to be drought-responsive in drought-tolerant and drought-sensitive plants; however, only a few miRNAs had identical response patterns between both plant groups (Fig. 5a, b). This result suggests that most drought-responsive miRNAs play important, yet different, roles in response to drought in different plants. For example, miR164 was downregulated by drought in drought-sensitive plants but not in drought-tolerant plants. This finding is similar to the downregulation of miR164 observed in *M. truncatula*^59^ and *P. trichocarpa*^13^ after drought treatment. Four out of six NAC genes (*OMTN2*, *OMTN3*, *OMTN4*, and *OMTN6*), the targets of miR164, negatively regulate drought resistance^60^, indicating that miR164 may contribute to the drought-sensitive phenotype. However, we did not identify any difference in the expression of miR164 under drought conditions in both plant groups, indicating that miR164 did not contribute to the drought-tolerant phenotype of drought-tolerant plants (Fig. 6 and Supplementary Table S7). In addition, miRn-249 was only induced by drought in drought-tolerant plants, with an expression level that was much higher in the same plant group after drought (Fig. 9 and Supplementary Table S7).

The genes associated with C3HC4-type RING finger participate in various plant biological processes^61^, indicating that miRn-249 might play a role in the drought tolerance of *Malus*. However, there was no difference in the expression levels of *Zinc finger* and miRn-249 under control conditions between both plant groups, indicating that *Zinc finger* may only be targeted by miRn-249 under drought conditions. In calli, the increased expression of miRn-249 could improve osmotic stress tolerance (Fig. 11), suggesting its positive roles in osmotic stress tolerance. However, the expression of its potential target, *Zinc finger* (*C3HC4-type RING finger*), was elevated slightly in transgenic MIRn-249 plants under control or stress conditions (Supplementary Fig. S4b), indicating that this target might not be cleaved by miRn-249 in the callus. Similarly, *SPL_2*, one of the targets of miR156p, may not be cleaved by miR156 in the calli. *Zinc finger* (*C3HC4-type RING finger*) may have been cleaved by miRn-249, as demonstrated by its lower expression in drought-tolerant plants after exposure to drought for 6 days (Fig. 10). We thus hypothesized that there might be other targets of miRn-249 in the calli, and that the positive role of miRn-249 in osmotic stress in calli was not via *Zinc finger* (*C3HC4-type RING finger*).

Some miRNAs that were not drought-responsive may still contribute to the drought tolerance of apple rootstock. The expression level of some novel miRNAs (miRn-157, miRn-158, and miRn-101) was lower in drought-tolerant than drought-sensitive plants under control or drought conditions (Fig. 9). The predicted target of miRn-157 is a member of the AP2/ERF and B3 domain-containing transcription factor family, which are involved in abiotic stress tolerance^62,63^. For example, the overexpression of *TSRF1*, an ERF transcription factor, improves the drought tolerance of rice^64^; thus, miRn-157 might contribute to the phenotype of drought-tolerant plants in response to drought. The functions of the predicted targets of both miRn-158 and miRn-101 are unknown, but the difference in the expression of both miRNAs between drought-sensitive and drought-tolerant plants under drought indicates that both molecules may participate in the drought response of apple rootstock. Alternatively, there may be an unknown mechanism for the improvement of drought tolerance in apple rootstock.

The stem-loop qRT-PCR results showed that miR156 expression in drought-tolerant plants was higher than that in drought-sensitive plants; however, miR156 was not induced by drought in either plant group (Fig. 9). This result diverges from those observed under drought conditions in other plant species, such as wheat^65^, where miR156 expression was induced. Indeed, our small RNA-seq results suggest that miR156 p/q/r/s transcripts were reduced under drought in drought-sensitive plants but remained stable in drought-tolerant plants (Fig. 6 and Supplementary Table S7). This phenomenon might be a result of several miR156 tags being detected simultaneously in the stem-loop qRT-PCR analysis, which might potentially affect each other.

Identifying differentially expressed miRNAs in both plant groups under drought conditions is critical to understand the genetic mechanisms underlying the drought tolerance of apple rootstocks. The GO enrichment analysis of predicted targets of differentially expressed miRNAs demonstrated that the enriched biological processes were mainly stress-related (Fig. 8). In wheat leaves, compared with the drought-sensitive genotype, drought-induced programmed cell death in drought-tolerant genotypes promoted higher levels of peroxidase, superoxide dismutase, catalase activities, and ascorbate content under drought stress^66^. Plant development can also contribute to drought tolerance. In sorghum, drought adaptation is associated with canopy development and root growth^67^. In molecular function, ubiquitin-protein ligase activity is associated with drought in *Arabidopsis*, where the expression of *RZFP34/CHYR1*, a ubiquitin E3 ligase, is significantly induced by drought. Loss-of-function and gain-of-function analyses of RZFP34/CHYR1 have suggested that RZFP34/CHYR1 promotes drought tolerance^68^.

Target prediction and qRT-PCR determined that *RNA polymerase II C-terminal domain phosphatase*, *WRKY33*, and *receptor kinase* are likely cleaved by miR395 (Figs. 9 and 10). WRKY33 protein plays an essential role in various biotic and abiotic stresses^69,70^. The homologous gene of receptor kinase in *Arabidopsis* is a leucine-rich repeat protein (LRR-RLK family member, AT4G23740), whose function in response to drought is currently unknown, but this protein may play a critical role in plant drought tolerance, consistent with other LRR-RLK family members. The overexpression of *OsSIK1*, an RLK gene, improved drought tolerance in rice. This finding is further supported by knockout transgenic plants showing the drought-sensitive phenotype^71^. Transcripts of miR395 were more abundant in drought-tolerant than drought-sensitive plants under control and drought conditions (Fig. 9). Thus, the downregulation of its target genes may contribute to drought tolerance.

SPL transcription factors are known miR156 targets across plant species^58,72,73^ and these molecules play central roles in plant growth and development^21^. We found the expression pattern of *SPL_4* and *SPL_6a* is opposite that of miR156, suggesting that miR156 negatively regulated *SPL_4* and *SPL_6a* under both control and drought conditions. *SPL_5* and *SPL_13a* were only downregulated in drought-tolerant plants after drought, suggesting that, under drought stress, these three SPLs may be important for a miR156-mediated drought response (Fig. 10). In *Arabidopsis*, SPL3, SPL4, and SPL5 (SPL 3/4/5) potentiate the FLOWERING LOCUS T (FT)-FD module in photoperiodic flowering^74^. Early flowering is an adaptive strategy for plants to escape drought^20^; hence, the differential expression of miR156 in both plant groups of *Malus* under drought indicated that miR156 may participate in the drought stress response through *SPL_4* and *SPL_5* in apple rootstocks. In tobacco, SPL_6 is critical for N TIR-NB-LRR receptor-mediated plant innate immunity^75^. Furthermore, the alleviation of drought tolerance can be pathogen-mediated. For example, infection with the Cucumber mosaic virus improves drought tolerance in *Capsicum annum*, *Solanum lycopersicum*, and *Nicotiana tabacum*^76^, indicating that the upregulation of miR156 may improve the drought tolerance of drought-tolerant plants by targeting *SPL6* in apple. In alfalfa, miR156 promotes drought tolerance by silencing *SPL_13*^77^. The downregulation of *SPL_13a* in drought-tolerant plants under drought suggests that miR156 may improve the drought tolerance of apple rootstock by silencing *SPL_13a* (Fig. 10). In addition, when MIR156p was overexpressed in apple calli, the expression levels of *SPL_6a*, *SPL_9, SPL_12*, and *SPL_13a* but not *SPL_2*, were significantly reduced under control or osmotic stress conditions (Fig. 11 and Supplementary Fig. S4), indicating that *SPL_2* might not be a target of miR156p in calli.

In summary, our study provides a foundation for further exploration of the candidate miRNAs and their target mRNAs associated with drought response. Although we verified the roles of miR156 and miRn-249 in osmotic stress tolerance in calli, we will characterize the roles of more miRNAs with contrasting expression patterns in both genotypes in response to drought. We will use multiple approaches to explore the molecular mechanisms of these miRNAs in response to drought in apple. Future work should experimentally demonstrate the interactions between miRNAs and their target genes and the mechanisms underlying their roles in apple rootstock drought response, which will be useful for breeding drought-resistant dwarfing rootstocks.

## Conclusions

We examined the drought tolerance of F_1_ progenies of R3 × *M. sieversii* and the expression patterns of miRNAs in response to drought in drought-tolerant and drought-sensitive plants. Relative to drought-sensitive plants, 67 miRNAs were differentially expressed in drought-tolerant plants under drought conditions. Under drought stress, 61 and 35 miRNAs were differentially expressed in drought-tolerant and drought-sensitive plants, respectively. Go analysis demonstrated that predicted target genes were predominately associated with response to stimulus, cellular process, and metabolic process. This work provides a foundation for further developing a comprehensive understanding of the molecular networks involving miRNAs and their targets in response to drought stress in apple.

## Supplementary information

Figure S1

Figure S2

Figure S3

Figure S4

Supplementary material.

Supplementary Figure Legends.
