# Supplementary material for: Genome-wide identification of drought-responsive microRNAs in two sets of Malus from interspecific hybrid progenies
Source: Hortic Res. 2019 Jun 8;6:75. doi: 10.1038/s41438-019-0157-z (PMC6555824; doi:10.1038/s41438-019-0157-z)
Supplement: Supplementary file 1 — Figure S1 [file 41438_2019_157_MOESM1_ESM.pdf]

a

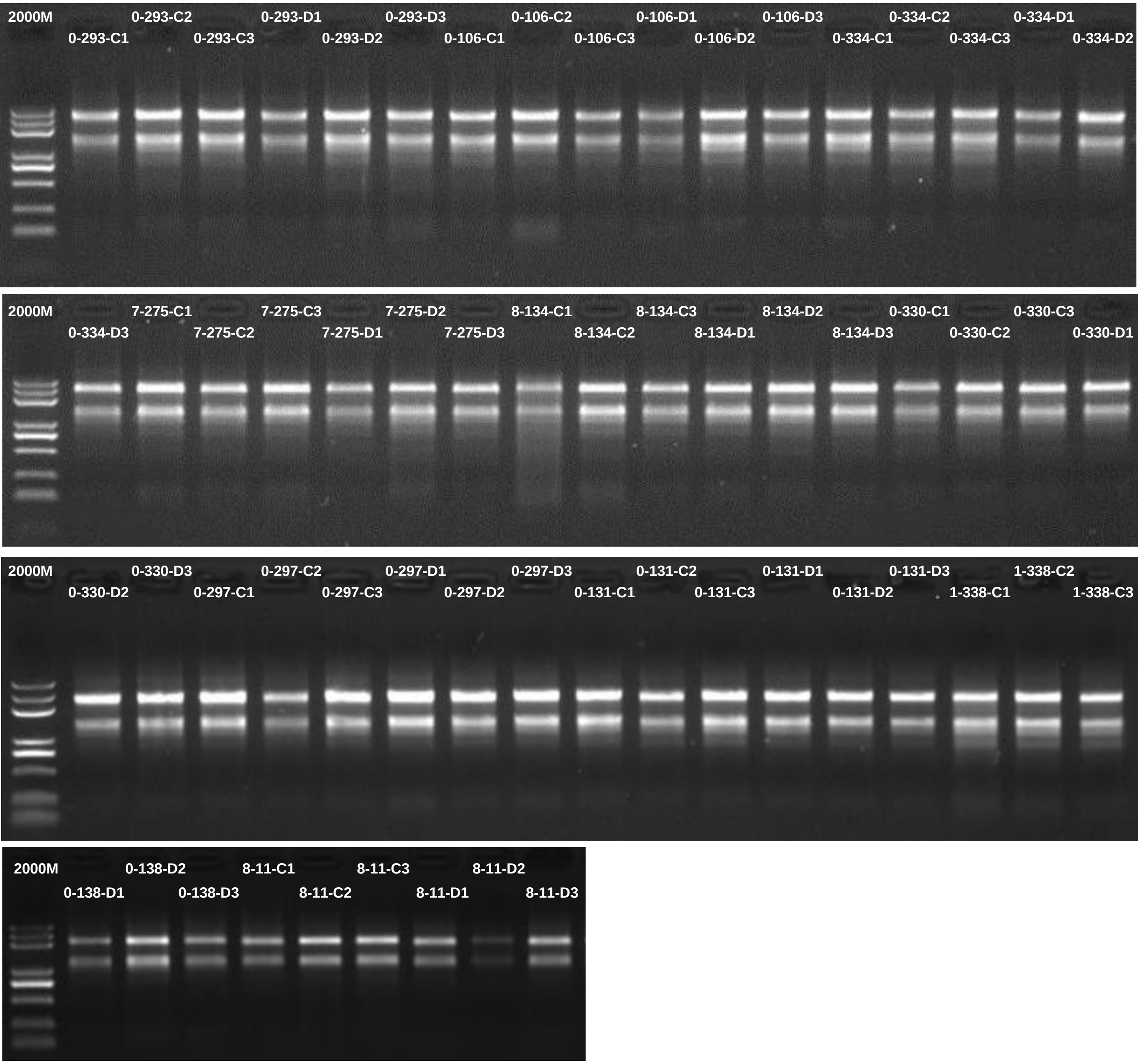

2000M
0-293-C1
0-293-C2
0-293-C3
0-293-D1
0-293-D2
0-293-D3
0-106-C1
0-106-C2
0-106-C3
0-106-D1
0-106-D2
0-106-D3
0-334-C1
0-334-C2
0-334-C3
0-334-D1
0-334-D2
2000M
0-334-D3
7-275-C1
7-275-C2
7-275-C3
7-275-D1
7-275-D2
7-275-D3
8-134-C1
8-134-C2
8-134-C3
8-134-D1
8-134-D2
8-134-D3
0-330-C1
0-330-C2
0-330-C3
0-330-D1
2000M
0-330-D2
0-330-D3
0-297-C1
0-297-C2
0-297-C3
0-297-D1
0-297-D2
0-297-D3
0-131-C1
0-131-C2
0-131-C3
0-131-D1
0-131-D2
0-131-D3
1-338-C1
1-338-C2
1-338-C3
2000M
0-138-D1
0-138-D2
0-138-D3
8-11-C1
8-11-C2
8-11-C3
8-11-D1
8-11-D2
8-11-D3

b

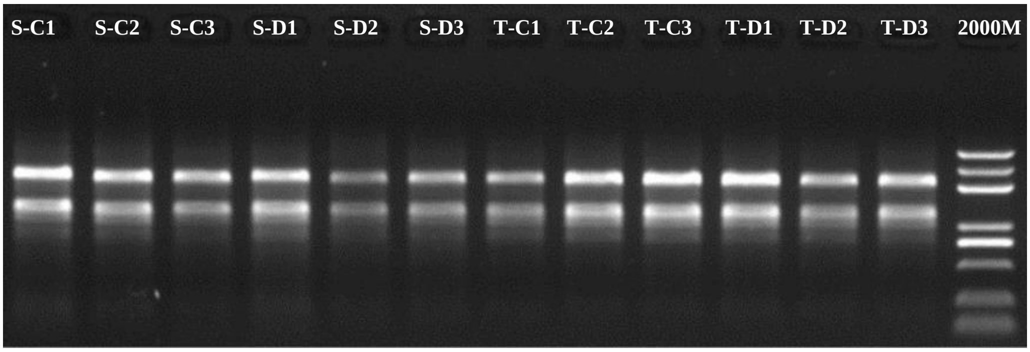

S-C1
S-C2
S-C3
S-D1
S-D2
S-D3
T-C1
T-C2
T-C3
T-D1
T-D2
T-D3
2000M

c

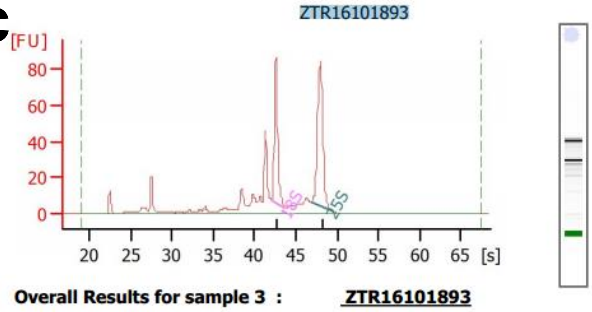

Overall Results for sample 3 : ZTR16101893

RNA Area:	512.2
RNA Concentration:	267 ng/µl
rRNA Ratio [25s / 18s]:	1.5
RNA Integrity Number (RIN):	7.3 (B.02.08)
Result Flagging Color:	
Result Flagging Label:	RIN: 7.30

Fragment table for sample 3 : ZTR16101893

Name	Start Time [s]	End Time [s]	Area	% of total Area
18S	42.07	43.68	85.1	16.6
25S	46.86	49.55	126.7	24.7

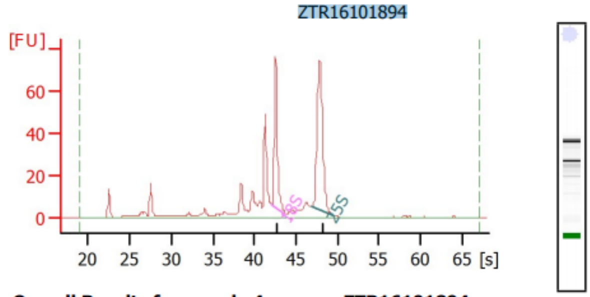

Overall Results for sample 4 : ZTR16101894

RNA Area:	428.5
RNA Concentration:	223 ng/µl
rRNA Ratio [25s / 18s]:	1.6
RNA Integrity Number (RIN):	7.3 (B.02.08)
Result Flagging Color:	
Result Flagging Label:	RIN: 7.30

Fragment table for sample 4 : ZTR16101894

Name	Start Time [s]	End Time [s]	Area	% of total Area
18S	42.08	43.73	73.7	17.2
25S	46.80	49.53	117.4	27.4

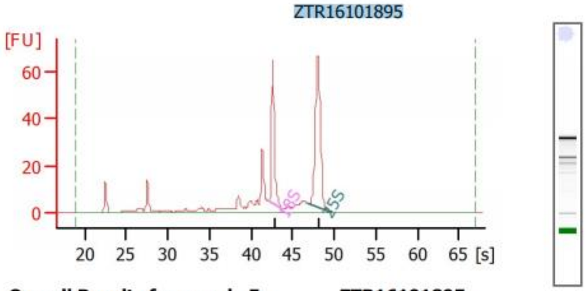

Overall Results for sample 5 : ZTR16101895

RNA Area:	326.9
RNA Concentration:	170 ng/µl
rRNA Ratio [25s / 18s]:	1.6
RNA Integrity Number (RIN):	7.8 (B.02.08)
Result Flagging Color:	
Result Flagging Label:	RIN: 7.80

Fragment table for sample 5 : ZTR16101895

Name	Start Time [s]	End Time [s]	Area	% of total Area
18S	42.18	43.74	59.7	18.3
25S	46.85	49.71	97.9	29.9

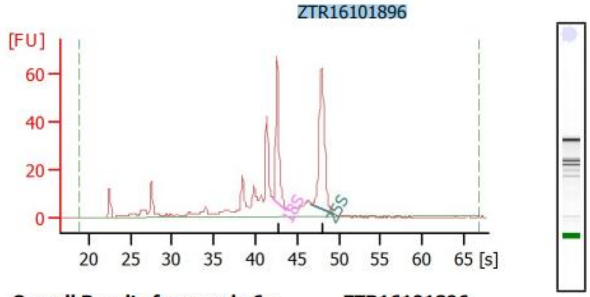

Overall Results for sample 6 : ZTR16101896

RNA Area:	437.8
RNA Concentration:	228 ng/µl
rRNA Ratio [25s / 18s]:	1.5
RNA Integrity Number (RIN):	6.8 (B.02.08)
Result Flagging Color:	
Result Flagging Label:	RIN: 6.80

Fragment table for sample 6 : ZTR16101896

Name	Start Time [s]	End Time [s]	Area	% of total Area
18S	42.14	43.74	65.0	14.8
25S	46.70	49.46	94.9	21.7

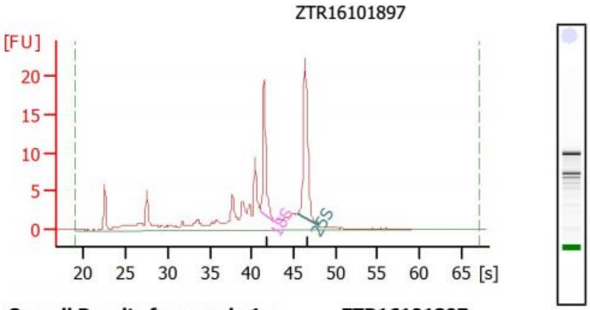

Overall Results for sample 1 : ZTR16101897

RNA Area:	136.9
RNA Concentration:	120 ng/µl
rRNA Ratio [25s / 18s]:	1.6
RNA Integrity Number (RIN):	7.1 (B.02.08)
Result Flagging Color:	
Result Flagging Label:	RIN: 7.10

Fragment table for sample 1 : ZTR16101897

Name	Start Time [s]	End Time [s]	Area	% of total Area
18S	41.10	42.66	17.3	12.6
25S	45.58	48.02	26.8	19.6

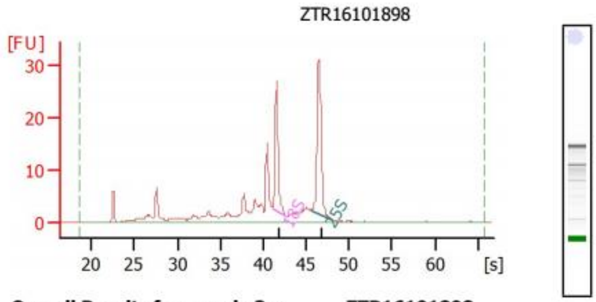

Overall Results for sample 3 : ZTR16101898

RNA Area:	169.6
RNA Concentration:	149 ng/µl
rRNA Ratio [25s / 18s]:	1.5
RNA Integrity Number (RIN):	7 (B.02.08)
Result Flagging Color:	
Result Flagging Label:	RIN:7

Fragment table for sample 3 : ZTR16101898

Name	Start Time [s]	End Time [s]	Area	% of total Area
18S	41.14	42.62	23.3	13.7
25S	45.67	48.05	35.6	21.0

ZTR16101899

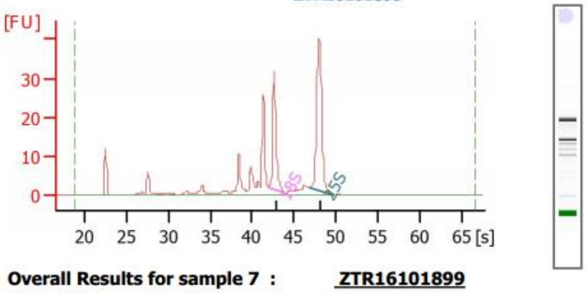

Overall Results for sample 7 : ZTR16101899

RNA Area:	192.3
RNA Concentration:	100 ng/µl
rRNA Ratio [25s / 18s]:	1.8
RNA Integrity Number (RIN):	7.5 (B.02.08)
Result Flagging Color:	
Result Flagging Label:	RIN: 7.50

Fragment table for sample 7 : ZTR16101899

Name	Start Time [s]	End Time [s]	Area	% of total Area
18S	41.95	43.98	32.8	17.0
25S	46.84	49.79	57.9	30.1

ZTR16101900

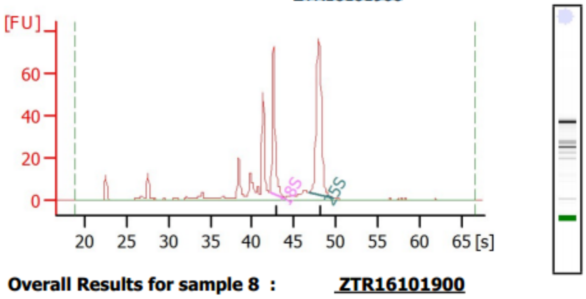

Overall Results for sample 8 : ZTR16101900

RNA Area:	370.3
RNA Concentration:	193 ng/µl
rRNA Ratio [25s / 18s]:	1.7
RNA Integrity Number (RIN):	7.5 (B.02.08)
Result Flagging Color:	
Result Flagging Label:	RIN: 7.50

Fragment table for sample 8 : ZTR16101900

Name	Start Time [s]	End Time [s]	Area	% of total Area
18S	42.15	43.89	66.2	17.9
25S	46.79	49.84	113.9	30.8

ZTR16101901

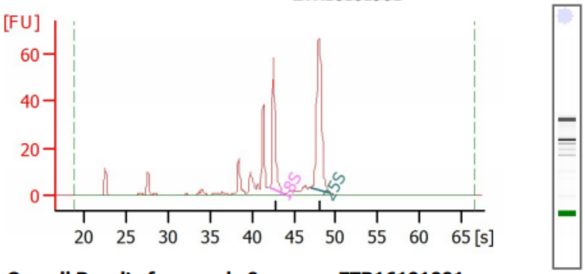

Overall Results for sample 9 : ZTR16101901

RNA Area:	270.5
RNA Concentration:	141 ng/µl
rRNA Ratio [25s / 18s]:	1.8
RNA Integrity Number (RIN):	7.7 (B.02.08)
Result Flagging Color:	
Result Flagging Label:	RIN: 7.70

Fragment table for sample 9 : ZTR16101901

Name	Start Time [s]	End Time [s]	Area	% of total Area
18S	42.10	43.79	53.4	19.7
25S	47.03	49.54	93.8	34.7

ZTR16101902

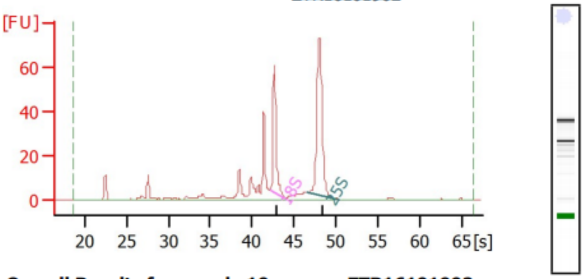

Overall Results for sample 10 : ZTR16101902

RNA Area:	307.1
RNA Concentration:	160 ng/µl
rRNA Ratio [25s / 18s]:	1.9
RNA Integrity Number (RIN):	7.8 (B.02.08)
Result Flagging Color:	
Result Flagging Label:	RIN: 7.80

Fragment table for sample 10 : ZTR16101902

Name	Start Time [s]	End Time [s]	Area	% of total Area
18S	42.16	43.80	57.2	18.6
25S	46.54	50.29	108.7	35.4

ZTR16101903

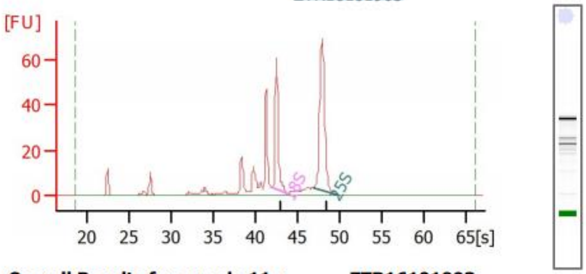

Overall Results for sample 11 : ZTR16101903

RNA Area:	327.5
RNA Concentration:	170 ng/µl
rRNA Ratio [25s / 18s]:	1.8
RNA Integrity Number (RIN):	7.3 (B.02.08)
Result Flagging Color:	
Result Flagging Label:	RIN: 7.30

Fragment table for sample 11 : ZTR16101903

Name	Start Time [s]	End Time [s]	Area	% of total Area
18S	42.07	43.85	54.4	16.6
25S	46.87	49.99	96.2	29.4

ZTR16101904

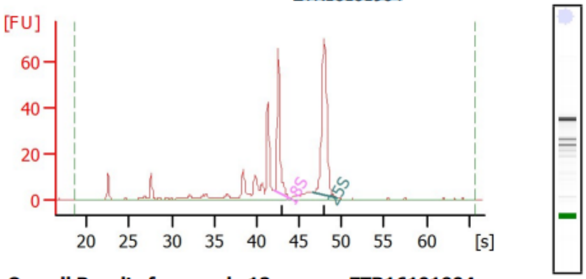

Overall Results for sample 12 : ZTR16101904

RNA Area:	314.2
RNA Concentration:	163 ng/µl
rRNA Ratio [25s / 18s]:	1.7
RNA Integrity Number (RIN):	7.4 (B.02.08)
Result Flagging Color:	
Result Flagging Label:	RIN: 7.40

Fragment table for sample 12 : ZTR16101904

Name	Start Time [s]	End Time [s]	Area	% of total Area
18S	42.14	43.81	58.9	18.7
25S	46.48	49.48	102.5	32.6
