# Supplementary figures and images for: Genome-wide identification of drought-responsive microRNAs in two sets of Malus from interspecific hybrid progenies
Source: Hortic Res. 2019 Jun 8;6:75. doi: 10.1038/s41438-019-0157-z (PMC6555824; doi:10.1038/s41438-019-0157-z)

**a**

miR156

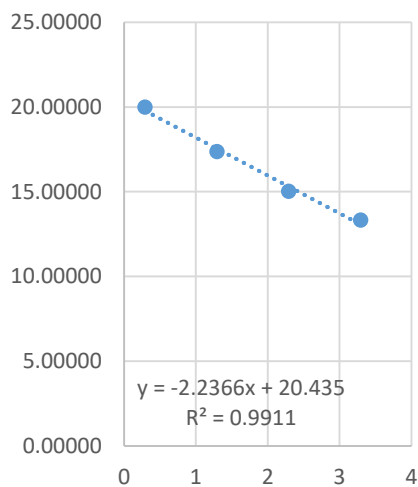

miR395

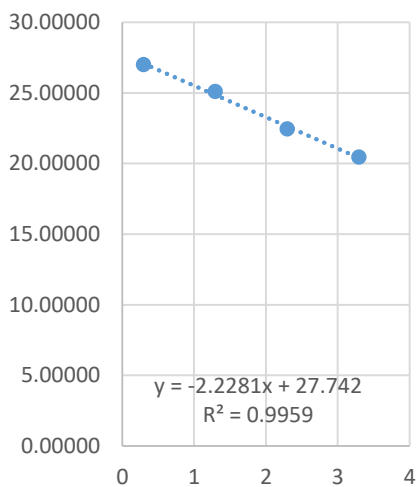

miR408a

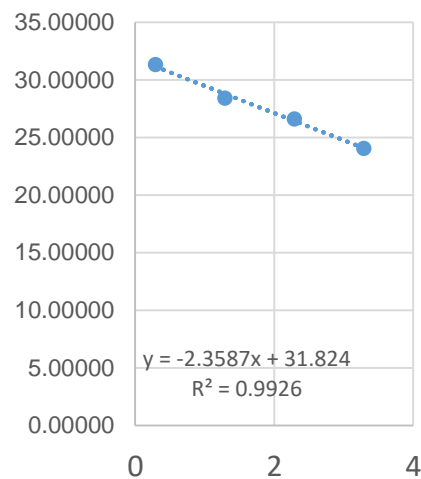

miR5225

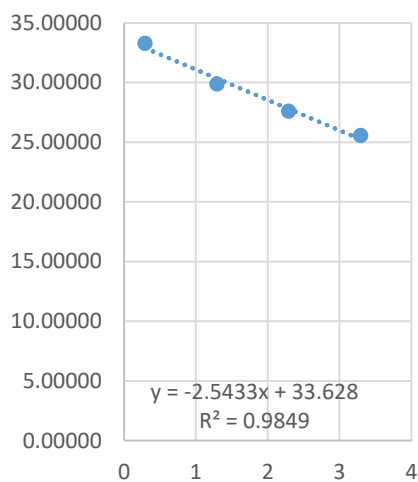

miRn-101

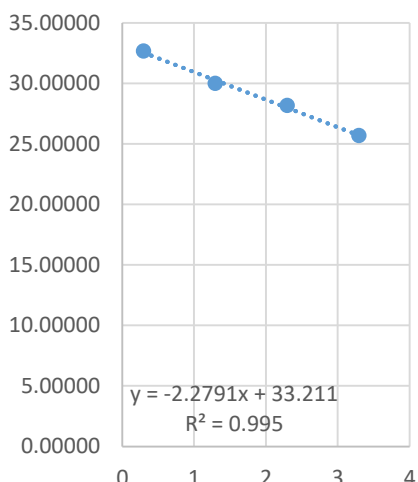

miRn-157

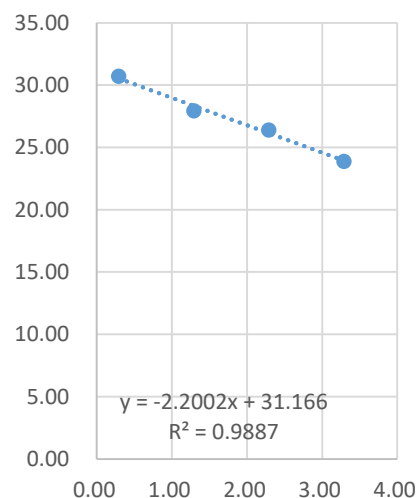

miRn-158

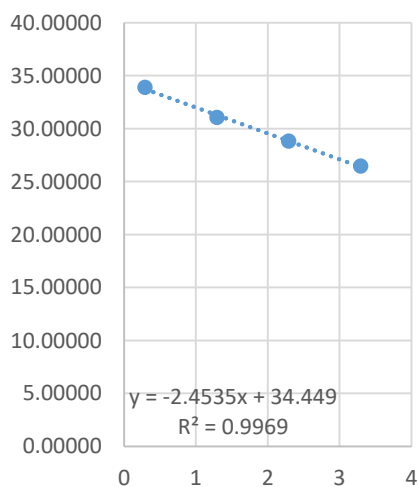

miRn-249

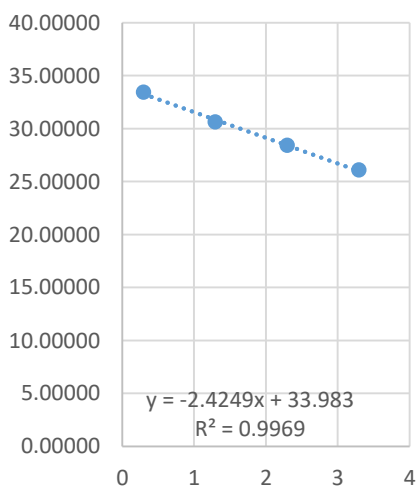

MDH

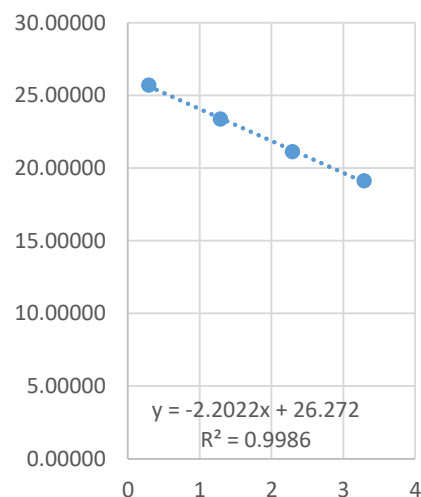

**b**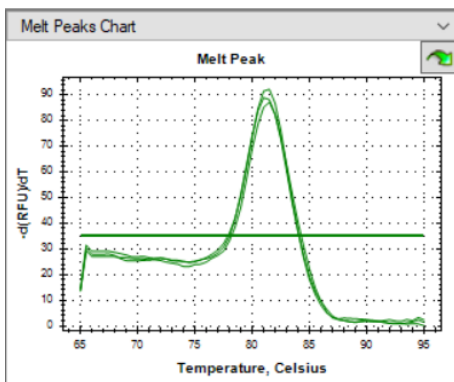**miR156**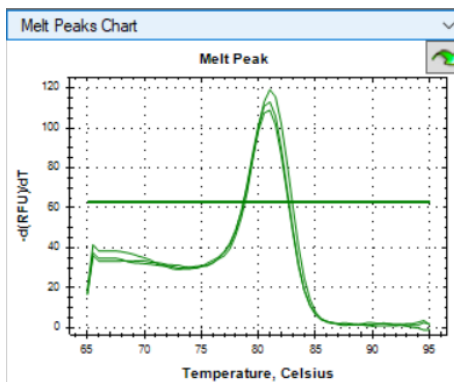**miR395**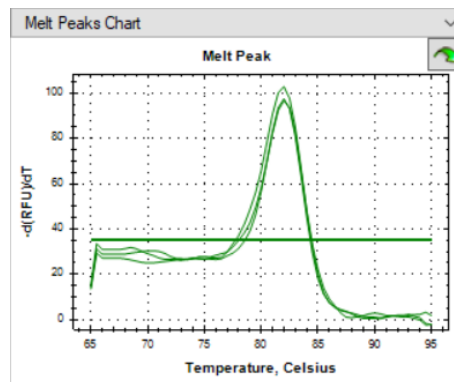**miR408a**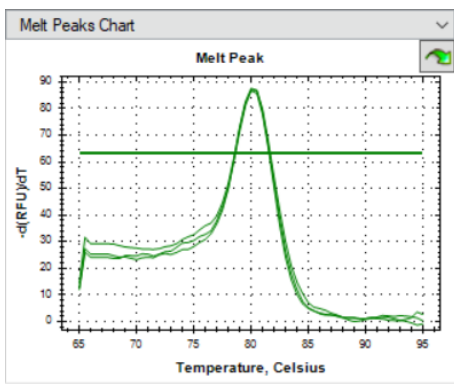**miR5225**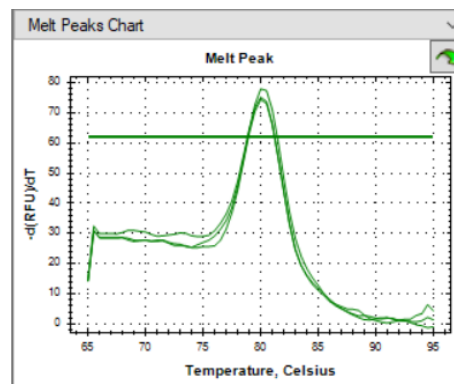**miRn-101**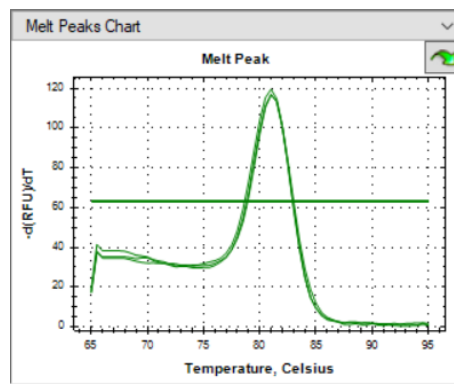**miRn-157**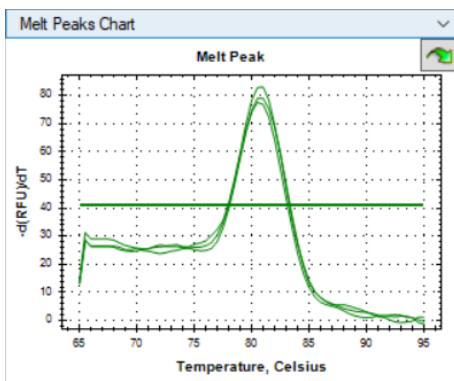**miRn-158**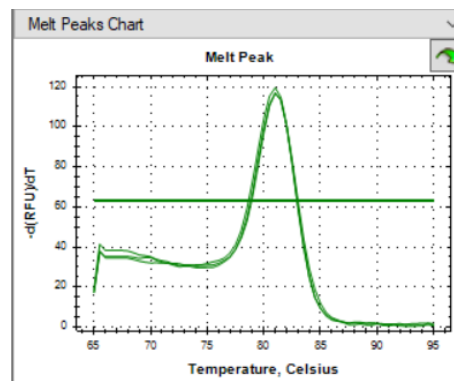**miRn-249**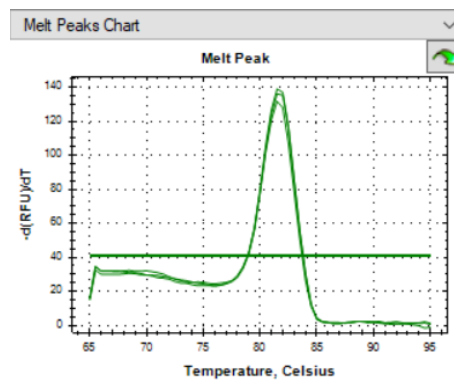**MDH**

Supplement: Supplementary file 2 — Figure S2 [file 41438_2019_157_MOESM2_ESM.pdf]
