# Supplementary material for: Genome-wide identification of drought-responsive microRNAs in two sets of Malus from interspecific hybrid progenies
Source: Hortic Res. 2019 Jun 8;6:75. doi: 10.1038/s41438-019-0157-z (PMC6555824; doi:10.1038/s41438-019-0157-z)
Supplement: Supplementary file 3 — Figure S3 [file 41438_2019_157_MOESM3_ESM.pdf]

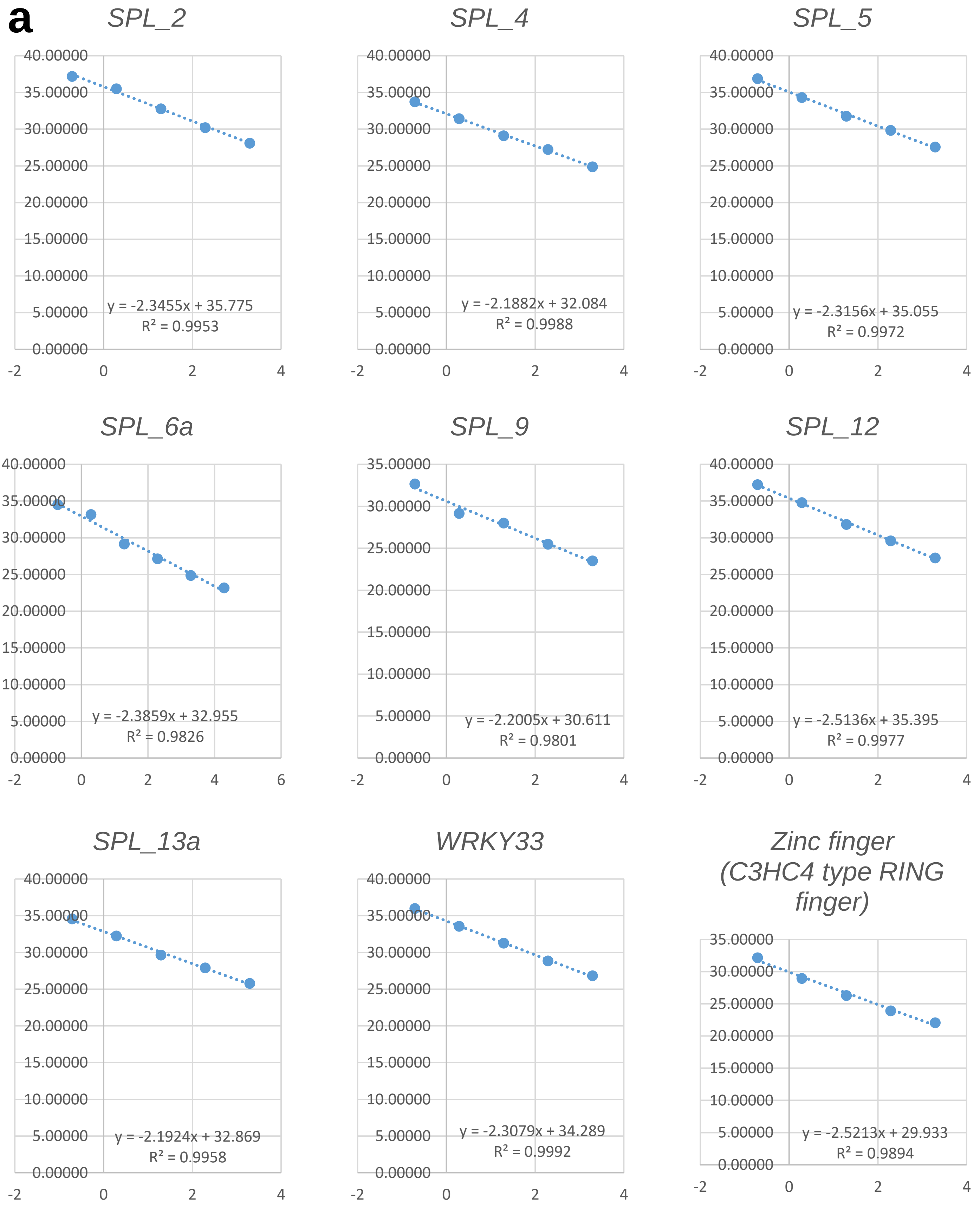
a
SPL_2
y = -2.3455x + 35.775
R² = 0.9953
SPL_4
y = -2.1882x + 32.084
R² = 0.9988
SPL_5
y = -2.3156x + 35.055
R² = 0.9972
SPL_6a
y = -2.3859x + 32.955
R² = 0.9826
SPL_9
y = -2.2005x + 30.611
R² = 0.9801
SPL_12
y = -2.5136x + 35.395
R² = 0.9977
SPL_13a
y = -2.1924x + 32.869
R² = 0.9958
WRKY33
y = -2.3079x + 34.289
R² = 0.9992
Zinc finger (C3HC4 type RING finger)
y = -2.5213x + 29.933
R² = 0.9894

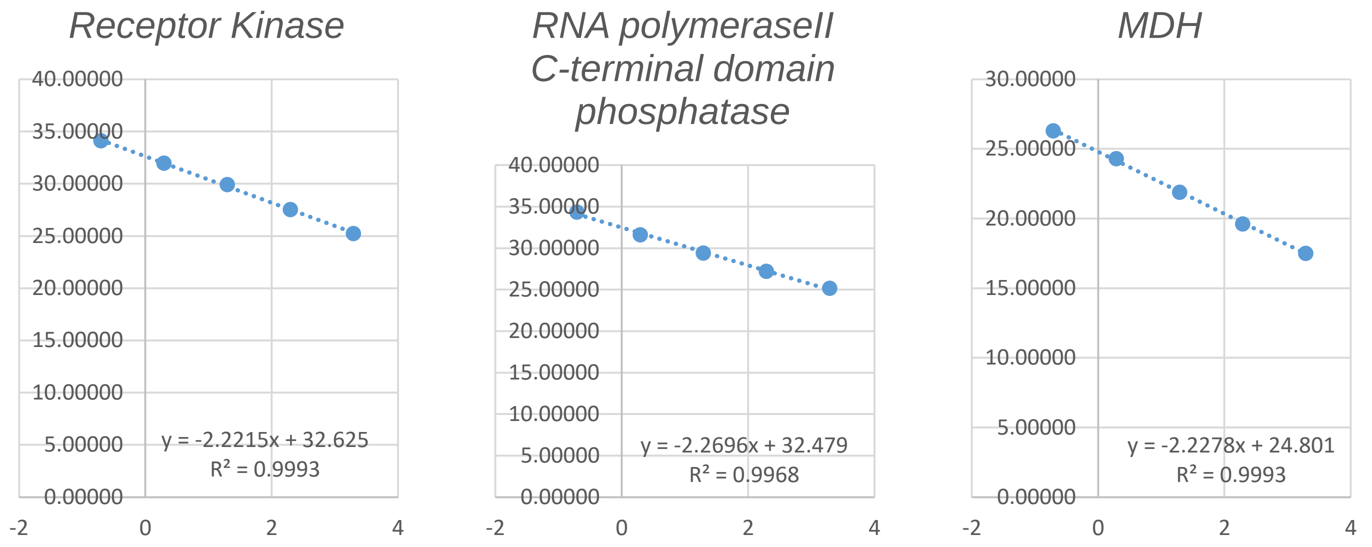

Receptor Kinase
y = -2.2215x + 32.625
R^2 = 0.9993
RNA polymeraseII C-terminal domain phosphatase
y = -2.2696x + 32.479
R^2 = 0.9968
MDH
y = -2.2278x + 24.801
R^2 = 0.9993

b

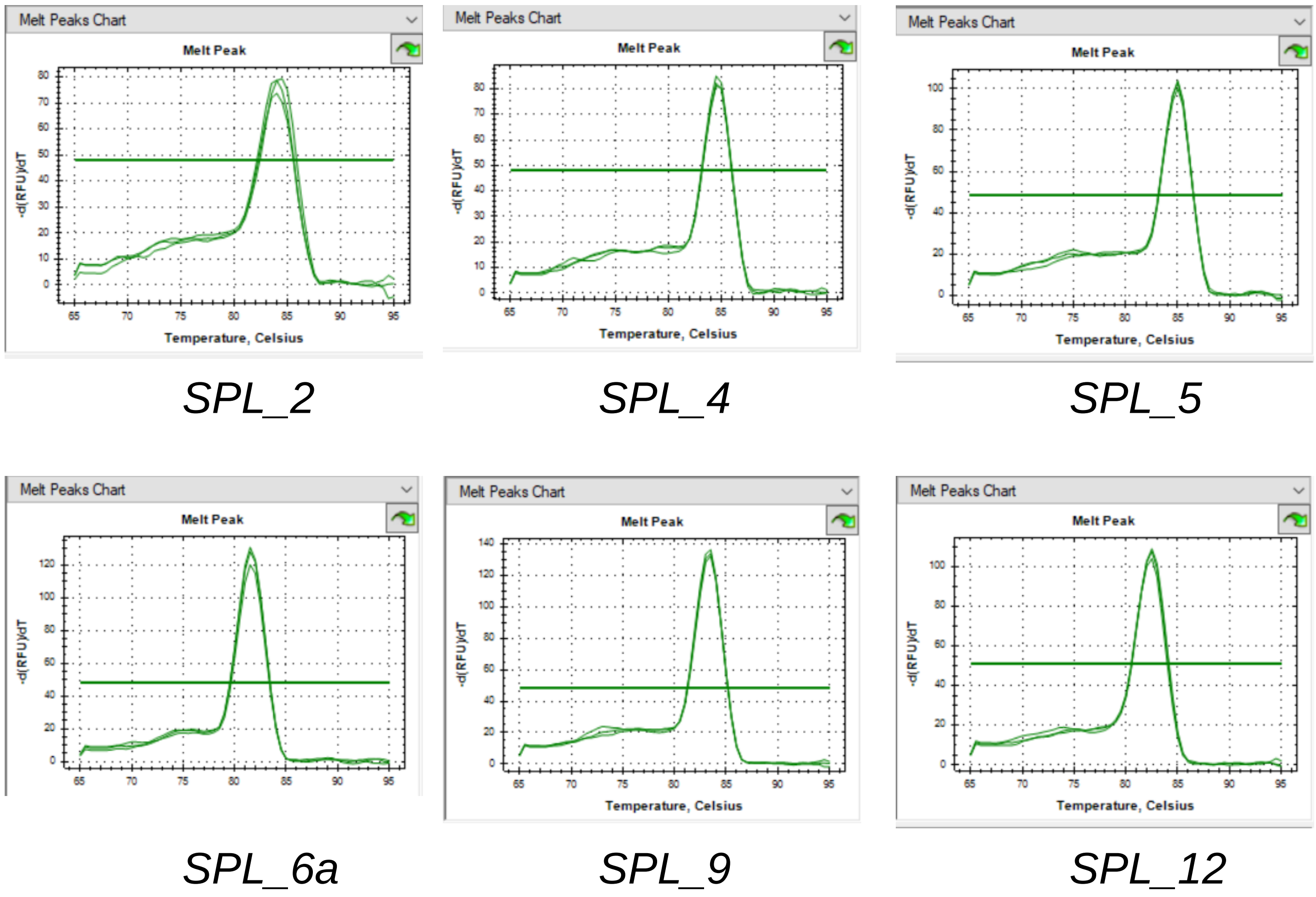

Melt Peaks Chart
Melt Peak
-d(RFU)/dT
Temperature, Celsius
SPL_2
SPL_4
SPL_5
SPL_6a
SPL_9
SPL_12

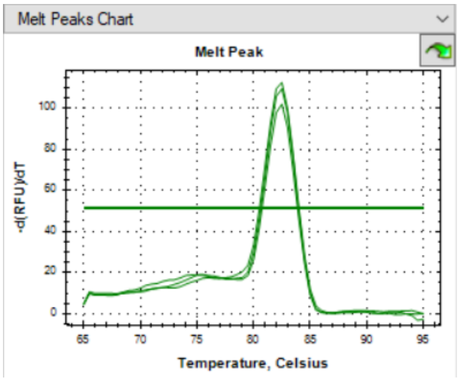

SPL_13a

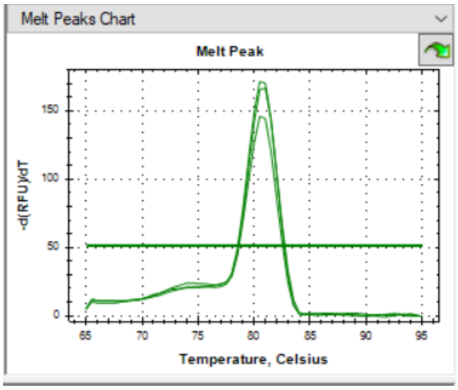

WRKY33

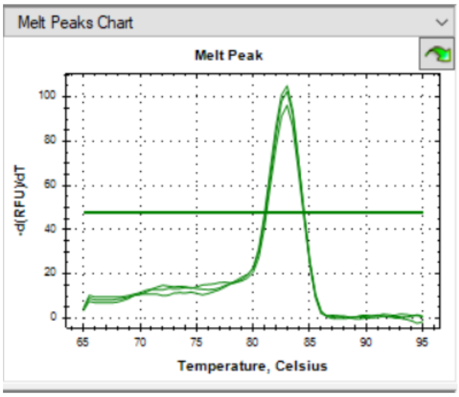

Zinc finger (C3HC4 type RING finger)

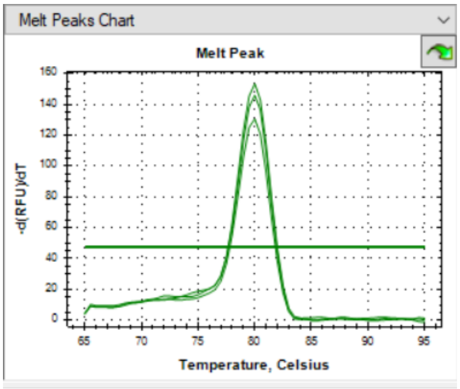

Receptor Kinase

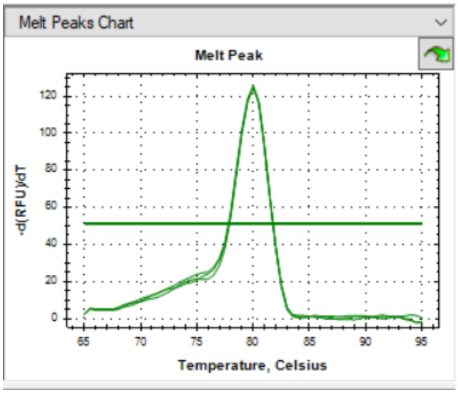

RNA polymeraseII C-terminal domain phosphatase

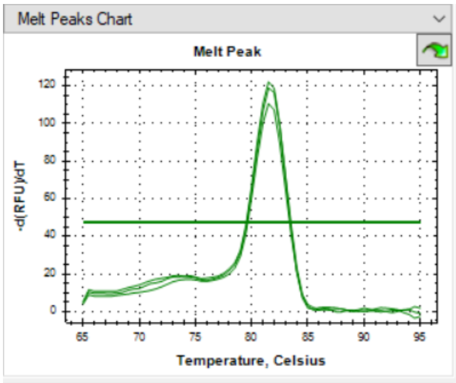

MDH
