# Supplementary material for: Genome-wide identification of drought-responsive microRNAs in two sets of Malus from interspecific hybrid progenies
Source: Hortic Res. 2019 Jun 8;6:75. doi: 10.1038/s41438-019-0157-z (PMC6555824; doi:10.1038/s41438-019-0157-z)
Supplement: Supplementary file 4 — Figure S4 [file 41438_2019_157_MOESM4_ESM.pptx]

## Slide 1
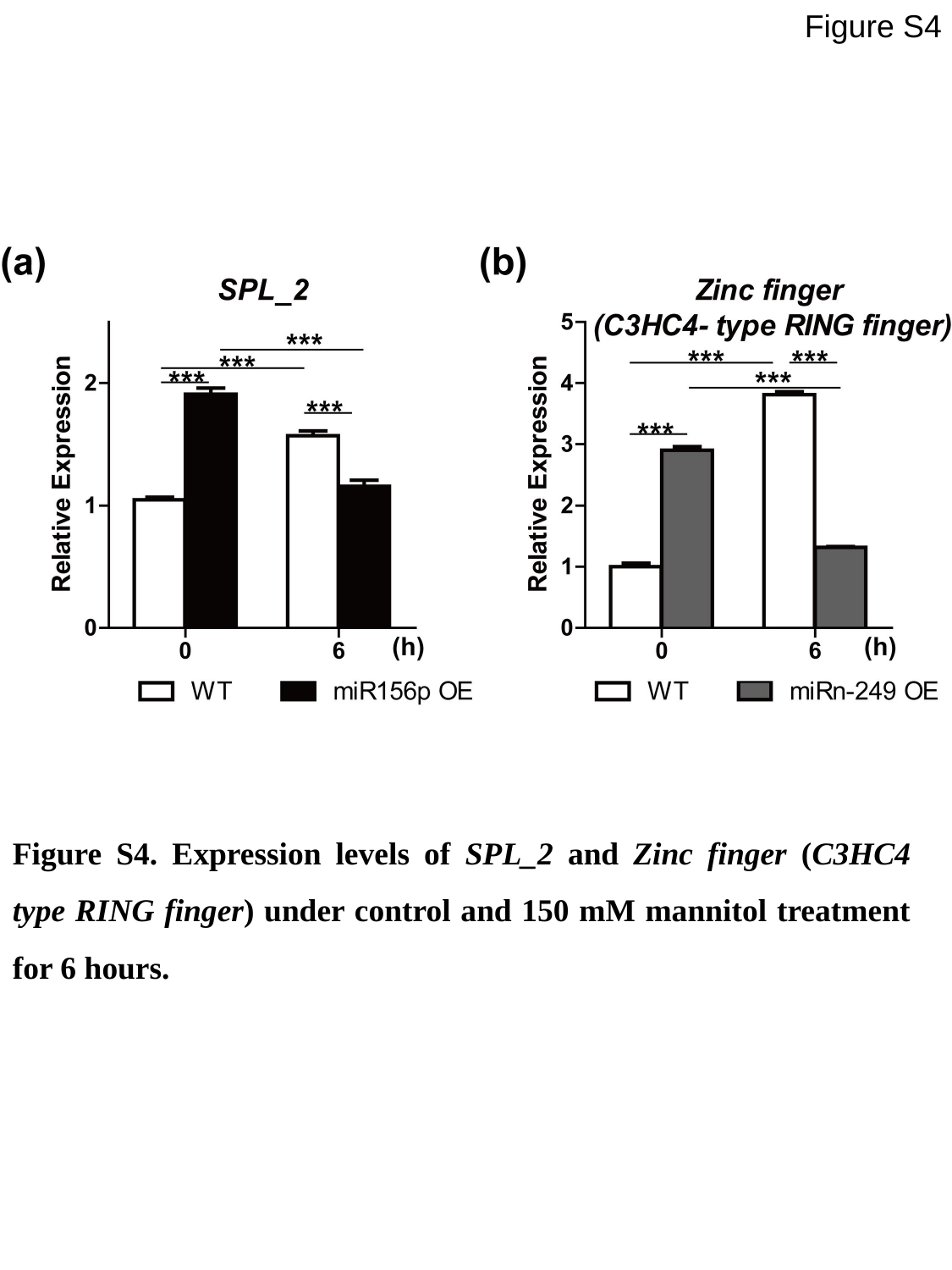

Figure S4
Figure S4. Expression levels of SPL_2 and Zinc finger (C3HC4 type RING finger) under control and 150 mM mannitol treatment for 6 hours.
