# Supplementary material for: Genome-wide identification of drought-responsive microRNAs in two sets of Malus from interspecific hybrid progenies
Source: Hortic Res. 2019 Jun 8;6:75. doi: 10.1038/s41438-019-0157-z (PMC6555824; doi:10.1038/s41438-019-0157-z)
Supplement: Supplementary file 6 — Supplementary Figure Legends. [file 41438_2019_157_MOESM6_ESM.pdf]

Figure S1. RNA quality controls information. (a) Agarose electrophoresis of extracted RNA for each biological replicate of F_1 progeny under control or drought treatment. (b) Agarose electrophoresis of pooled RNA. (c) Detection of RNA integrity using the Agilent 2100. ZTR16101893 to ZTR16101904 represent drought-sensitive plants under control conditions (1, 2, and 3), drought-sensitive plants under drought conditions (1, 2, and 3), drought-tolerant plants under control conditions (1, 2, and 3), and drought-tolerant plants under drought conditions (1, 2, and 3), respectively.

Figure S2. Efficiency (a) and melting curve (b) of primers used for miRNA expression determined by stem-loop qRT-PCR.

Figure S3. Efficiency (a) and melting curve (b) of primers used for miRNA target genes detected by qRT-PCR.

Figure S4. Expression levels of *SPL_2* and *Zinc finger* (*C3HC4 type RING finger*) under control and 150 mM mannitol treatment for 6 h.
